# Distinct Expansion of Group II Introns During Evolution of Prokaryotes and Possible Factors Involved in Its Regulation

**DOI:** 10.3389/fmicb.2022.849080

**Published:** 2022-02-28

**Authors:** Masahiro C. Miura, Shohei Nagata, Satoshi Tamaki, Masaru Tomita, Akio Kanai

**Affiliations:** ^1^Institute for Advanced Biosciences, Keio University, Tsuruoka, Japan; ^2^Systems Biology Program, Graduate School of Media and Governance, Keio University, Fujisawa, Japan; ^3^Faculty of Environment and Information Studies, Keio University, Fujisawa, Japan

**Keywords:** group II intron, intron-encoded protein, bioinformatics, genomic signatures, prokaryotic genomes

## Abstract

Group II introns (G2Is) are ribozymes that have retroelement characteristics in prokaryotes. Although G2Is are suggested to have been an important evolutionary factor in the prokaryote-to-eukaryote transition, comprehensive analyses of these introns among the tens of thousands of prokaryotic genomes currently available are still limited. Here, we developed a bioinformatic pipeline that systematically collects G2Is and applied it to prokaryotic genomes. We found that in bacteria, 25% (447 of 1,790) of the total representative genomes had an average of 5.3 G2Is, and in archaea, 9% (28 of 296) of the total representative genomes had an average of 3.0 G2Is. The greatest number of G2Is per genome was 101 in *Arthrospira platensis* (phylum Cyanobacteriota). A comprehensive sequence analysis of the intron-encoded protein (IEP) in each G2I sequence was conducted and resulted in the addition of three new IEP classes (U1–U3) to the previous classification. This analysis suggested that about 30% of all IEPs are non-canonical IEPs. The number of G2Is per genome was defined almost at the phylum level, and at least in the following two phyla, Firmicutes, and Cyanobacteriota, the type of IEP was largely associated as a factor in the G2I increase, i.e., there was an explosive increase in G2Is with bacterial C-type IEPs, mainly in the phylum Firmicutes, and in G2Is with CL-type IEPs, mainly in the phylum Cyanobacteriota. We also systematically analyzed the relationship between genomic signatures and the mechanism of these increases in G2Is. This is the first study to systematically characterize G2Is in the prokaryotic phylogenies.

## Introduction

Introns are present in various forms across the three domains of life, bacteria, archaea, and eukaryota. The best known is the pre-mRNA intron in the eukaryotic nuclear genome, which is spliced by spliceosomes (Papasaikas and Valcarcel, 2016). Group I introns (G1Is) and group II introns (G2Is) have been found in prokaryotes (both bacteria and archaea) and eukaryotic organelles (Edgell et al., 2011). Bulge–helix–bulge (BHB) introns are also inserted into some pre-tRNAs and pre-rRNAs in archaea, and into some pre-tRNAs in eukaryotes, and are enzymatically spliced by endoribonucleases and RNA ligases (Tang et al., 2002; Yoshihisa, 2014). We developed a computational program to comprehensively extract tRNA introns from predominantly archaeal genomes and detected tRNA genes that are disrupted in various ways (Sugahara et al., 2008; Fujishima et al., 2009). In the context of this research, and based on the theory that G2Is contributed to the process that established the nucleus of eukaryotes (Martin and Koonin, 2006) and the facts that the tertiary structure of spliceosome is similar to that of G2Is (indicating an evolutionary relationship between the spliceosome and G2I) (Haack and Toor, 2020) and a huge number of complete prokaryotic genome sequences is currently available, we decided to systematically analyze the G2I, which comprises an intron, a ribozyme, and a mobile element in prokaryotes.

Standard G2I RNAs are divided structurally into domains I–VI based on their RNA secondary structures (Ferat and Michel, 1993). Some of these secondary-structural domains have special functions. For example, the domain I region contains the target-binding sites for G2I transposition, and the domain V region acts as the center of ribozyme activity in splicing (Pyle, 2016). Although G2I is a ribozyme, intron-encoded proteins (IEPs) are encoded in domain IV (Ferat and Michel, 1993). Each IEP consist of a reverse transcriptase (RT) domain and other domains [e.g., DNA-binding domain and endonuclease (En) domain] (Zimmerly and Semper, 2015). During transposition, the DNA-binding domain and En domain bind to the target region of DNA and break the bottom DNA strand (the DNA strand into which G2I is not integrated, respectively) (Lambowitz and Belfort, 2015). However, in some types of IEP, the En domain is not structurally included in the IEP and is not essential for transposition (Garcia-Rodriguez et al., 2019). Thus, the G2I RNA and IEP form a complex, and the splicing and transposition activities are conducted by the cooperative action of each functional domain of the G2I RNA and IEP (Haack and Toor, 2020). G2Is are classified based on the similarity of the secondary structures of their RNAs and the similarity of the amino acid sequences of their IEPs (Novikova and Belfort, 2017). Some G2Is do not have an IEP [open reading frame (ORF)-less type] (Simon et al., 2008). In rare cases, non-standard G2Is have also been reported in which the RNA secondary structure is degenerate, the protein encoded by the ORF is largely lacking due to frameshift, or the encoded protein is a homing endonuclease. However, at least some of the non-standard G2Is have splicing activity (Salman et al., 2012; McNeil et al., 2014).

Attempts have been made to computationally identify regions corresponding to G2Is in genomic DNA data. In the early 2000s, Zimmerly collected bacterial and archaeal G2Is using a sequence-similarity-search-based method and published it as a database (Dai and Zimmerly, 2002, 2003). Since then, the G2I sequence published by that group has been used as a query by other groups, and G2Is have been collected continuously from genomic sequences (Abebe et al., 2013; Titov et al., 2019; Sonbol and Siam, 2021). These analyses have shown the diversity of G2Is, the numbers of G2Is per genome, and their insertion positions in genomes. Toro and Nisa-Martínez collected various reverse transcriptases, including the IEPs in G2Is, and showed that the G2Is can be classified into 13 types based on the similarities of their RT domains (Toro and Nisa-Martinez, 2014). More recently, Toro et al. (2019) constructed a large dataset of reverse transcriptases containing over 4,000 G2I IEPs. It has also been shown that in most bacteria, the number of G2Is per genome is ≤ 3 and rarely exceeds 10 (Leclercq et al., 2011). Moreover, G2Is often nest within the intergenic regions that are not harmful to the host bacterium or within other mobile genetic elements, including G2Is or related genes, whereas some G2Is have been reported to disrupt essential genes (Waldern et al., 2020).

As mentioned above, numerous studies have provided much insight into G2Is with bioinformatic analyses. However, while the number of prokaryote genomes registered in public databases is continuing to increase, the bioinformatic analyses to address the overall picture of the G2Is in the database are still limited. In this study, we developed a bioinformatic pipeline that systematically collects G2Is and applied it to the complete prokaryotic genomes (approximately 15,000) currently available. By classifying the collected G2Is, we clarified the possible existence of new G2I groups based on the IEP sequences, and the spread of fragmented non-canonical IEPs in prokaryotes. The phylogenetic information on the collected G2Is and the host genomes allowed us to establish the detailed distributions of G2Is in bacteria and archaea. G2Is were present across many bacterial phyla (25% of the total bacterial genomes) and were concentrated in the phylum Halobacterota (class Methanomicrobia) in the archaea (9% of the total archaeal genomes). The number of G2Is was generally defined at the phylum level, but there were many cases in which the numbers differed significantly among closely related species. A dramatic increase in G2Is in prokaryotes occurred with combinations of specific IEP types and bacterial taxa, and these increases may be associated with particular genomic signatures, such as transcription terminators and GC skew.

## Results and Discussion

### Comprehensive Extraction of Group II Introns From Prokaryotic Genomes

In an attempt to understand the exact distribution of G2Is in prokaryotic genomes, it is necessary to comprehensively identify the genomic region of each G2I. We first constructed a new bioinformatic pipeline that comprehensively identifies G2Is in prokaryotic genomes. This program allowed us to identify genomic sequences containing the most conserved RNA secondary structures of G2Is, domains V and VI, and at least parts of domains I–IV. Another feature of the pipeline is that it can handle G2Is with or without IEPs. A summary of the pipeline is given below (Supplementary Figure 1A). In step 1, the three major domains of the G2Is were extracted: (i) the RT domain of the IEP, (ii) RNA domains V and VI, and (iii) RNA domains I–IV. The RNA secondary structure model registered in the Rfam database was used to extract domains V and VI, and parameters were established to identify 341 of the 347 (98%) G2Is classified as “Eubacterial,” “Archaeal,” or “ORF-less” in the Database for Bacterial Group II Introns (Zbase) (Candales et al., 2012). In step 2, when the RT domain occurred within the 1,300 bases of domain V, the sequence was interpreted as the IEP-containing type of G2I (Supplementary Figure 1B). In step 3, if an RT domain was not considered to be part of G2I in step 2, and domains I–IV were within the 1,300 bases of domain V, the sequence was interpreted as a no-IEP-type or ORF-less G2I (Supplementary Figure 1C). Therefore, when setting the threshold for the distances between domains in steps 2 and 3, we calculated the length of each region of the G2Is in Zbase and in a previous study (Toro and Nisa-Martinez, 2014). The threshold was set to 1,300 bases because the distance between the RT domain and domain V was ≤ 1,300 bases in 99% of the identified G2Is. For the ORF-less-type G2Is, the threshold was set to 1,300 bases based on the same criterion.

To verify the pipeline, we selected 12 typical prokaryotes that are considered to have G2Is and 18 typical prokaryotes that are not considered to have any G2Is by referring to the Zbase, and used the program to extract G2Is from these genomes (Supplementary Table 1). The results indicated that in 18 species for which no G2I was reported, our program detected no G2Is, i.e., there were no false positive results. Furthermore, our program detected 98% of the G2Is with IEPs and the ORF-less-type G2Is in which no RNA secondary-structural domain was deleted, as shown in Supplementary Table 1. These two types of G2Is accounted for 80% of the 434 G2Is of prokaryotes registered in Zbase, and we consider that the majority of G2Is in prokaryotic genomes belong to one or other of these types. Similarly, most previous studies that collected G2Is from genomic data targeted either one of these two types. This demonstrates that our program can effectively collect more data than was achieved in previous studies. However, in the following cases, there was a slight discrepancy between the results of our program and the prior studies. First, if multiple G2Is had a nested structure, called a “twintron” (Pfreundt and Hess, 2015), our program could not detect the outer G2I. This situation was found in the genome of *Thermosynechococcus elongatus* and *Wolbachia* endosymbiont (false negative case #1). Additionally, our program did not detect the G2I-like sequences in *T. elongatus* that lacked IEPs and most of domains I–IV (false negative case #2). Although the program detected G2Is that are considered to be pseudogenes because the IEPs have frameshift mutations, these sequences were not registered in Zbase (false positive case #1). This result was classified as a false positive here, but as explained later, it is classified as a G2I with a non-canonical IEP in this paper. This situation was detected in the genomes of *Enterococcus faecalis* and *Methanosarcina mazei*. In *Methanococcoides burtonii*, a region containing domains V and VI was duplicated before and after the IEP in all four G2Is. This resulted in the presence of two genes per G2I (false positive case #2). We undertook a large-scale data analysis of the main G2Is mentioned above, including the G2I-like sequences but excluding the G2Is with exceptional structures.

### Number of Group II Introns Largely Dictated by the Host Phylum

Using our program, 13,041 G2Is were detected in 14,506 bacterial genomes downloaded from the National Center for Biotechnology Information (NCBI) Reference Sequence (RefSeq) database. Of these, 12,153 were G2Is with IEPs and 888 were G2Is without IEPs. In analyzing the systematic distribution of species with G2Is, we focused on 1,790 representative genomes to eliminate the bias in the species registered in the database (see section “Materials and Methods”). These 1,790 genomes contained 2,381 G2Is, of which 2,100 had IEPs and 281 did not. Of these 1,790 genomes, 447 (about 25% of the total) contained one or more G2Is (Supplementary Table 2A). In previous studies, the details of the method used and the types of G2I targeted differed between research groups, and the abundance of G2Is in bacteria has been reported to be 12–35% (Leclercq et al., 2011; Candales et al., 2012; Waldern et al., 2020). A previous study claimed that the prevalence of G2Is in bacteria is approximately 25% of the total genomes (Candales et al., 2012), and our results support this observation. The species in which G2Is were found and their locations in these genomes are summarized in Supplementary Table 3. Information on the 14,506 bacterial genomes used in this study is summarized in Supplementary Table 4.

**FIGURE 1:**
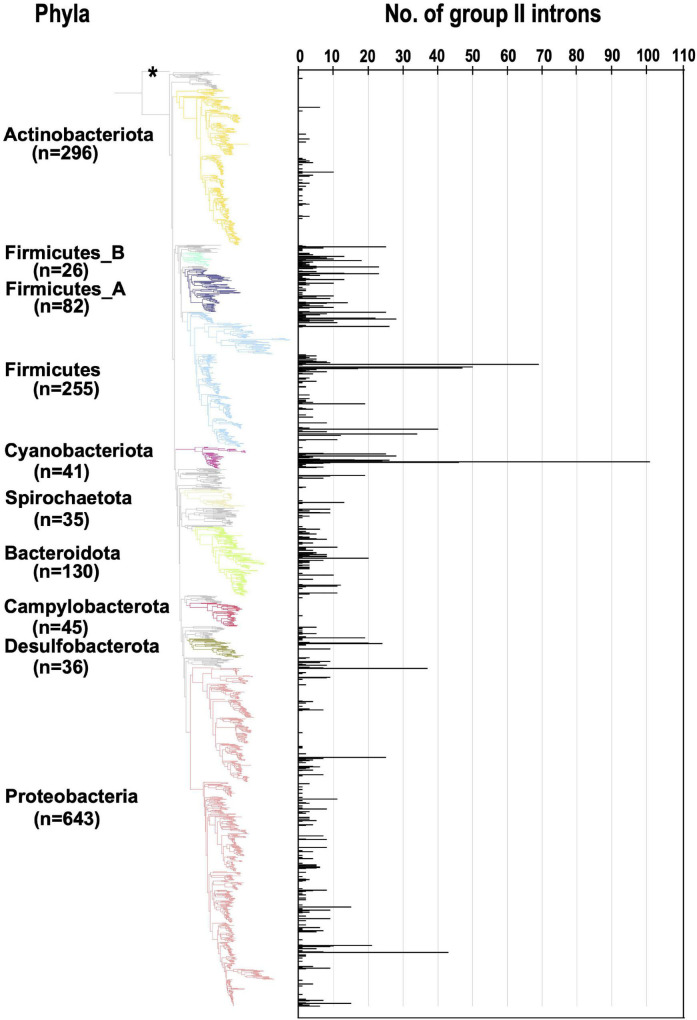
Increase in the number of G2Is in specific bacterial genomes belonging to each bacterial phylum. The numbers of G2Is in representative complete bacterial genomes (1,774 genomes) are shown. Bacterial phyla are shown on the left and each corresponding branch on the bacterial phylogenic tree is colored. The numbers in bracket represents the number of genomes in each phylum. The position of the outgroup [*Candidatus Saccharibacteria* oral taxon TM7x (RefSeq assembly accession: GCF_000803625.1)] is indicated by the asterisk.

We next analyzed the number of G2Is per bacterial genome (Figure 1). In the 447 bacterial genomes containing G2Is, the average number ± standard deviation of G2Is was 5.3 ± 8.8, and the median was 2, suggesting considerable variability. Here, 54 genomes (∼12% of the total) had ≥ 10 G2Is, and the following three phyla contained the majority of these genomes: Firmicutes, 27 genomes; Cyanobacteriota, 7 genomes; and Proteobacteria, 8 genomes. The largest number of G2Is was in *Arthrospira platensis* (RefSeq assembly accession: GCF_000210375.1), which had 101 G2Is. In Cyanobacteriota, *Thermosynechococcus elongatus* and *Trichodesmium erythraeum* are also reported to have large numbers of G2Is (Nakamura et al., 2002; Pfreundt et al., 2014; Xu et al., 2016), and our analysis supports this observation. In contrast, in Actinobacteriota and Campylobacterota, most genomes had very few G2Is, and few genomes had ≥ 10 G2Is. Based on these results, we infer that the distribution of G2Is differs among phyla, and that the numbers of introns increased rapidly in specific bacterial phyla. We also analyzed the relationship between the number of G2Is and the genome size as a factor that might explains the differences in the species distributions and the numbers of G2Is across species of bacteria. The results showed that the correlation coefficient between the number of G2Is and genome size was low (*R*^2^ = 0.003 or 0.002), so no relationship was detected (Supplementary Figure 2A). The possibility of a relationship between the number of G2Is and genome size was also examined for each bacterial phylum, but no significant relationship was found in any phylum (Supplementary Figure 2B).

### Group II Introns Present in Large Numbers Have Specific Intron-Encoded Protein Types

**FIGURE 2:**
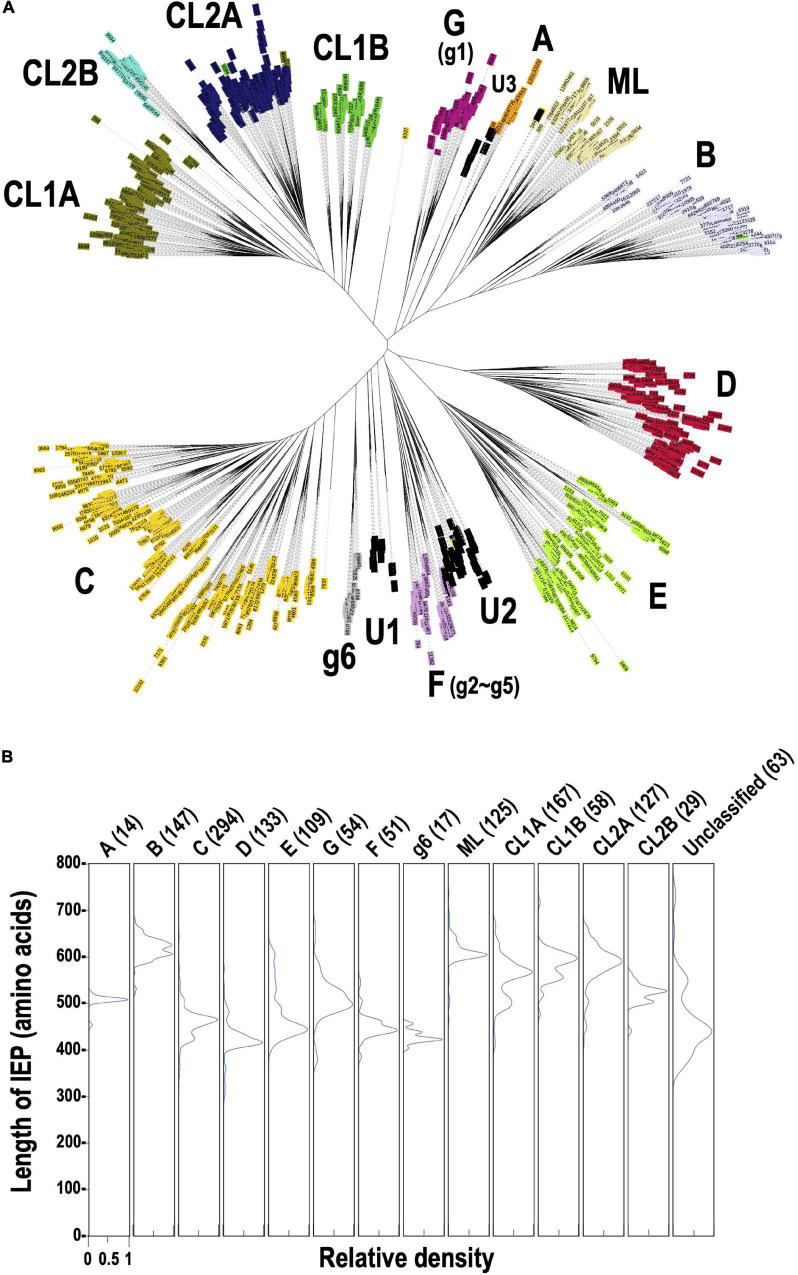
Phylogeny of the prokaryotic IEPs detected in this study. **(A)** An unrooted phylogenetic tree of representative IEP sets (1,949 proteins) in bacterial G2Is. The representative IEP sets were selected based on a similarity analysis (see section “Materials and Methods,” Prediction of IEP Sequences). The types of IEP are: A (bacterial-A, orange), B (bacterial-B, lavender), C (bacterial-C, yellow), D (bacterial-D, red), E (bacterial-E, light green), G [g1] (bacterial-G [g1], violet), F [g2–g5] (bacterial-F [g2–g5], plum), g6 (bacterial-g6, silver), ML (light yellow), CL1A (olive), CL1B (green), CL2A (blue), and CL2B (turquoise). U1–U3 (black) are newly identified clusters that were previously annotated as “unclassified.” **(B)** Distribution of the amino acid lengths of each canonical IEP. The peak relative density is set as 1.0 in each case.

To clarify why the number of G2Is per genome differs for each taxon, we undertook an analysis of the types of IEPs present in the G2Is. As described previously, 12,153 IEPs were extracted from 14,506 bacterial genomes. Figure 2A shows a phylogenetic tree constructed from 1,949 sequences of representative IEPs obtained with CD-HIT from 12,153 IEPs. Among the IEPs we collected, there were sequences that were interrupted by a stop codon and sequences that were clearly lacking some functional domains. These incomplete sequences accounted for about 30% of the total 1,949 IEP sequences used to construct the phylogenetic tree (Supplementary Figure 3). Regardless of the species or type of IEP, these “non-canonical” IEPs were widely distributed in the bacterial phylogeny (Supplementary Figures 4, 5). In contrast, in some cases, because nucleotide sequences similar to the corresponding region of each canonical IEP occur after the stop codon, it is possible that some non-canonical IEP sequences encode complete IEPs, expressed by read-through or frameshift mechanisms. The types of IEPs classified on this phylogenetic tree well reflect the IEP types in previous studies (Toro and Martinez-Abarca, 2013; Toro and Nisa-Martinez, 2014). We also found that the IEP sequences that were considered “unclassified” in the previous study clustered at three phylogenetic positions, and designated them U1, U2, and U3 on the current phylogenetic tree. U1, U2, and U3 are not monophyletic, but each has different sequence characteristics from the neighboring IEPs on the phylogenetic tree. For example, compared with the amino acid sequences of the bacterial-F (g2–g5)-type IEPs, most of the U1- and U2-type IEPs lack part of the RT domain (Supplementary Figure 6). Compared with the bacterial-A-type IEPs, U3 tends to lack a part of the RT domain and the DNA-binding domain is elongated (Supplementary Figure 7). These results suggest that the U1–U3 lineages constitute new phylogenetic subtypes, at least. More recently, Toro et al. (2019) constructed a large reverse transcriptase dataset containing 4,338 G2I IEPs. Using the 4,338 G2I IEP sequences described in that paper, we performed a comprehensive similarity analysis with our 1,392 IEPs that do not contain non-canonical IEPs and identified each IEP type in the dataset of Toro et al. (2019). The results showed that the proportion of each type of IEP across the bacteria in their dataset, including U1, U2, and U3, was quite similar to our proportions, even when we analyzed their dataset using our method (the correlation coefficient was calculated to be 0.96). Therefore, their dataset supports our classification and provides further examples. However, because our data (Figure 2A) also include information on non-canonical IEPs, they provide new information in this context.

Figure 2B shows the distribution of sequence lengths of the canonical ORFs for each IEP lineage group. The ORF lengths can be roughly classified into two groups: in one, they are approximately 600 amino acids long (bacterial-B, ML, CL1A, CL1B, and CL2A) and in the other, they are approximately 400–500 amino acids long (bacterial-A, C, D, E, G, F, g6, unclassified), depending on the presence or absence of the En domain. The CL2B-type IEP has an En domain, but the overall length is slightly reduced because the region of the DNA-binding domain is shorter than in other CL-type IEPs. When the distribution of the lengths of the ORFs of non-canonical IEPs is considered, many IEP types have a wide range of length and some very short sequences of about 150–350 amino acids. These are fragmentary IEPs, in which at least part of some domain(s) is not present (Supplementary Figure 8A). By contrast, among non-canonical IEPs, there are several sequences with the same lengths as canonical IEPs (Supplementary Figure 8B). These are sequences in which a relatively long ORF occurs due to a shift in the reading frame, although the same domain structure is not maintained. We are the first to undertake a comprehensive analysis of non-canonical IEPs in this way. Note that because each ORF sequence was selected to be as long as possible when the ORF was extracted, the methionine codon upstream from the exact translation start position can be used, and the sequence length may be overestimated.

**FIGURE 3:**
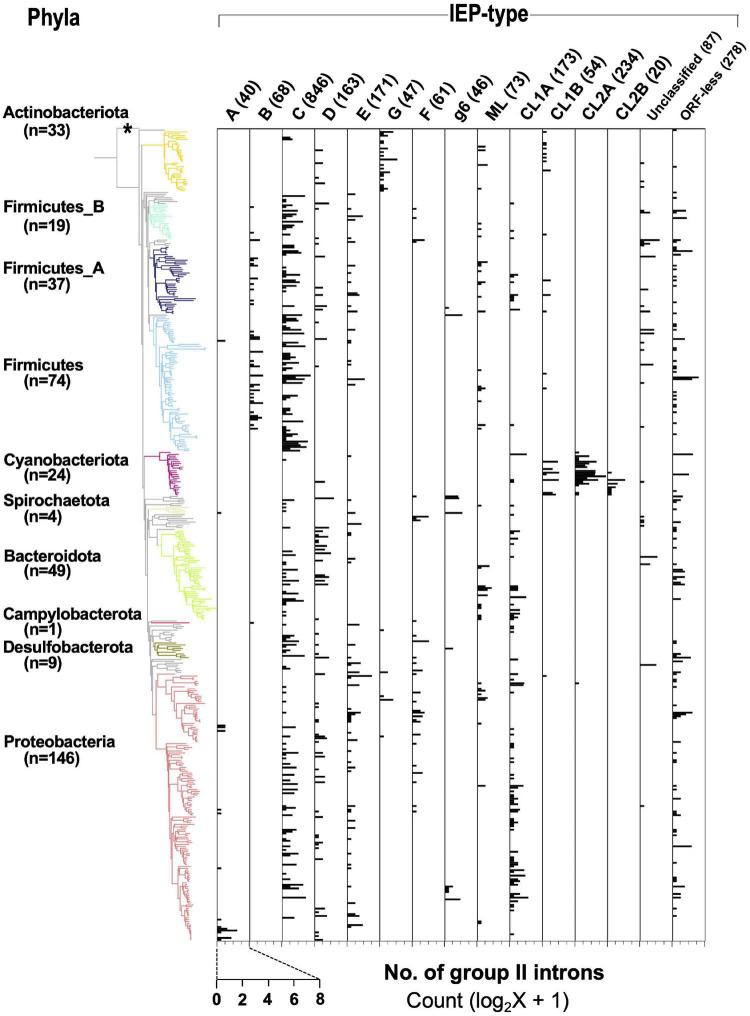
Distribution of types of IEPs in the bacterial phylogeny. A bacterial phylogenetic tree was constructed from 443 representative bacterial genomes whose genome contain G2I(s) (see section “Materials and Methods”), and *Candidatus Saccharibacteria* oral taxon TM7x (RefSeq assembly accession: GCF_000803625.1) was used as the outgroup. See the legends of Figures 1, 2 for details. The horizontal axis represents the number of G2Is for each IEP type.

The distribution of IEP types was examined in the bacterial genomes with G2Is. For this purpose, we used 443 of the 447 genomes with G2Is among the representative genomes of bacteria, excluding four genomes not used in the phylogenetic tree database (Figure 3 and Supplementary Table 2A). The IEP types corresponded well to specific bacterial phyla, such as bacterial B to Firmicutes, G[g1] to Actinobacteriota, CL2A to Cyanobacteriota, and CL2B to Cyanobacteriota, whereas bacterial-C, -D, and -E and CL1A are widely distributed throughout the bacterial phyla. The number of G2Is with bacterial-C-type IEPs was high in many species. This result is consistent with a previous study in which more than half of the IEPs found in 1,435 bacteria were of the bacterial-C type (Waldern et al., 2020). Number of G2Is by IEP type in each genome is summarized in Supplementary Table 5.

We investigated the kinds of IEP types in increasing G2Is from the following two perspectives: (i) genomes containing many G2Is (here, the top 25 genomes, or 1.4% of the total) were used; and (ii) 130 genomes in the phylum Cyanobacteriota, where species rich in G2Is are found, for which full-length genomes were available. First, using the 25 genomes with the highest number of G2Is, we analyzed the relationships between species with increasing G2Is and the IEP types. Supplementary Figure 9 shows that genomes belonging to Firmicutes tended to encode bacterial-C-type IEPs, and genomes belonging to Cyanobacteriota tended to encode CL-type IEPs. Furthermore, the number of G2Is with bacterial-C-type IEPs increased in phyla other than Firmicutes. However, CL-type G2Is were significantly increased only in the genomes belonging to Cyanobacteriota, except for one genome, *Vibrio campbellii_A*. Therefore, the two most strongly increasing IEP types differed in their distributions. Bacterial-E, unclassified, and ORF-less G2Is also increased in certain genomes. As characteristics of host bacteria in which G2Is increase, it has been reported that an obligate endosymbiotic bacterium, which is difficult to culture alone, and a thermophilic bacterium have a large number of G2Is (Mohr et al., 2010; Leclercq et al., 2011). In Supplementary Figure 9, *Orientia tsutsugamushi* is an obligate endosymbiont, and the five species *Symbiobacterium thermophilum*, *Thermoanaerobacter wiegelii*, *Natranaerobius thermophilus*, *Thermobacillus composti*, and *Thermosynechococcus elongatus* are thermophiles. By contrast, species with large numbers of bacterial-C-type G2Is include some that are not classified as obligate symbiotic bacteria or thermophiles. Therefore, other factors may also be involved in the increase in G2Is.

Next, we analyzed the relationship between the IEP types and the number of G2Is in the phylum Cyanobacteriota. This phylum contains species with the largest numbers of G2Is per genome, and information on the genomes of 130 cyanobacterial genomes, including non-representative genomes, is available. The results suggested that the phylum Cyanobacteriota includes species without G2Is and species with more than 100 G2Is, and that the constituent species differ greatly in their numbers of G2Is (Supplementary Figure 10). Particularly large numbers of G2Is were found in the family Phormidiaceae, which includes *Arthrospira platensis*. In this family, species in the genus *Arthrospira* have approximately 30–100 G2Is. However, in the family Phormidiaceae, only *Planktothrix agardhii_A* has fewer than 10 G2Is, which differs markedly from other species in this family. Therefore, the number of G2Is even varies among closely related species. Moreover, most of the genomes in Cyanobacteriota with one or more G2Is have CL-type G2Is. Based on these observations, although there is a mechanism that supports the increase of CL-type G2Is in Cyanobacteriota, factors other than the IEP type may also be thought to cause this variability in the number of G2Is, even among closely related species.

### Archaeal Group II Introns Are Concentrated in the Phylum Halobacterota

**FIGURE 4:**
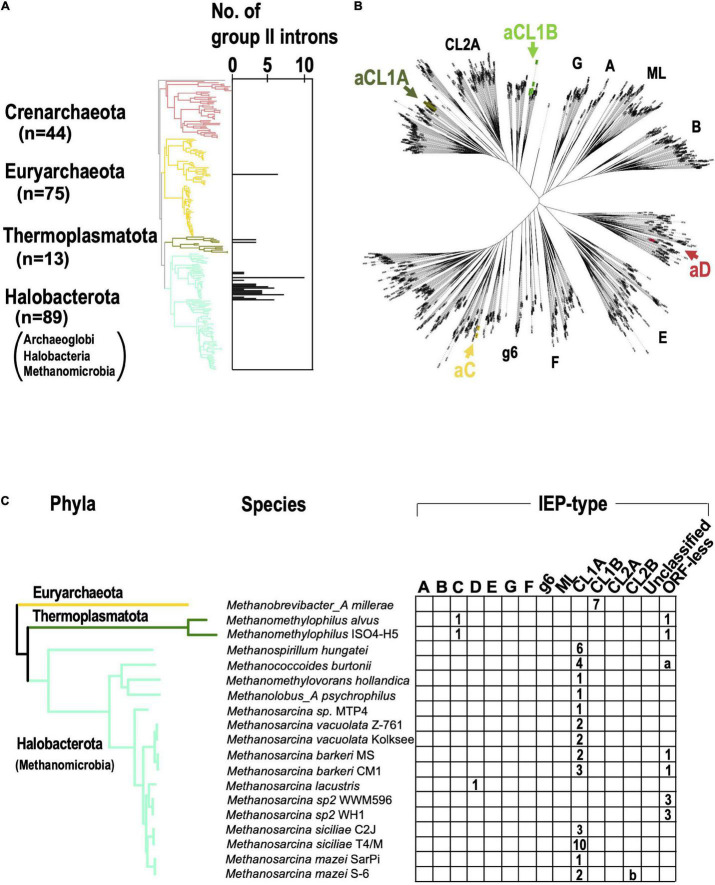
G2Is are increased in certain archaeal phyla. **(A)** Increases in numbers of G2Is in specific archaeal phyla. The numbers of G2Is in complete archaeal genomes (222 genomes) are shown. Archaeal phyla are shown on the left and each corresponding branch on the archaeal phylogenic tree is colored. The numbers in brackets represent the number of genomes in each phylum. **(B)** Positions of archaeal IEPs on an unrooted phylogenetic tree of the representative IEP sets (see Figure 2A in details). aCL1A (archaeal CL1A), aCL1B (archaeal CL1B), aC (archaeal-C), and aD (archaeal-D). **(C)** Distribution of types of IEPs in the archaeal phylogeny. The distribution of the types of IEPs in 19 archaeal genomes that contain G2I(s) is shown. Numbers of G2I(s) per IEP type are also shown in each box. a: Analysis of the *Methanococcoides burtonii* genome with our pipeline incorrectly detected four ORF-less G2Is. These were parts of CL1A-type G2Is. b: Analysis of the *Methanosarcina mazei* S-6 genome with our pipeline incorrectly detected one G2I classified as the CL2B type, because the G2I was divided by a transposase. A detailed analysis revealed that it was a CL1A-type G2I.

When the G2I search pipeline was applied to archaeal genomes, we found that 28 of 296 genomes had 84 G2Is (about 9% of the total archaeal genomes; Supplementary Table 2B). At the phylum level, 23% of the genomes in Halobacterota and 15% of the genomes in Thermoplasmatota had G2Is, but most other phyla had no G2Is (Supplementary Table 2B). Under the NCBI taxonomy, Halobacterota is grouped into three classes, Archaeoglobi, Halobacteria, and Methanomicrobia, and G2Is were detected only in the class Methanomicrobia. Of the 84 genomes with G2Is, 67 (approximately 80%) were concentrated in the phylum Halobacterota (i.e., class Methanomicrobia). The result indicates that G2Is occur in only limited archaeal phyla, in contrast to the spread of G2Is in bacterial phyla. The abundance of G2Is in Methanomicrobia may reflect previous studies that found that large amounts of bacterial genes have been transferred into the archaeal phylum containing Methanomicrobia during the evolution of prokaryotes (Nelson-Sathi et al., 2012, 2015). We note that the current analysis was limited to the near-complete archaeal genomes in the database, so Asgard archaea have not been analyzed, while G2Is have also been found in these species in previous studies (Vosseberg and Snel, 2017). We also calculated the number of G2Is per genome in archaea and mapped them onto the archaeal phylogenetic tree obtained from the Genome Taxonomy Database (Figure 4A). In the 28 archaeal genomes containing G2Is, the average number ± standard deviation of G2Is was 3.0 ± 2.2, and the median was 2. The phylogenetic tree also confirmed that archaeal G2Is are concentrated in Halobacterota. In bacteria, some species have large numbers of G2Is, sometimes ≥ 20, but in archaea, the maximum number of G2Is per genome is 10. On the IEP phylogenetic tree shown in Figure 4B, the archaeal IEPs are distributed into four clades (bacterial-C, -D, CL1A, and CL1B), and form monophyletic groups distinct from those of the bacterial IEPs (Figure 4B). CL1A is the commonest IEP type in archaea (Figure 4C), and among the 19 archaeal genomes with G2Is, 13 have CL1A-type G2Is, and *Methanosarcina siciliae* T4/M has 10 CL1A-type G2Is. With the comprehensive analysis undertaken in this study, no significant increase in bacterial-C-type G2Is, as seen in bacteria, was detected in archaea. These results are consistent with previous studies that showed a limited increase in G2Is within archaea as compared with bacteria (Simon et al., 2008). Therefore, in the subsequent analysis, we focused on bacteria and further investigated the factors behind the remarkable increases in G2Is.

### Group II Introns With Specific Intron-Encoded Protein Types Tend to Integrate Just After Rho-Independent Transcription Terminators

**FIGURE 5:**
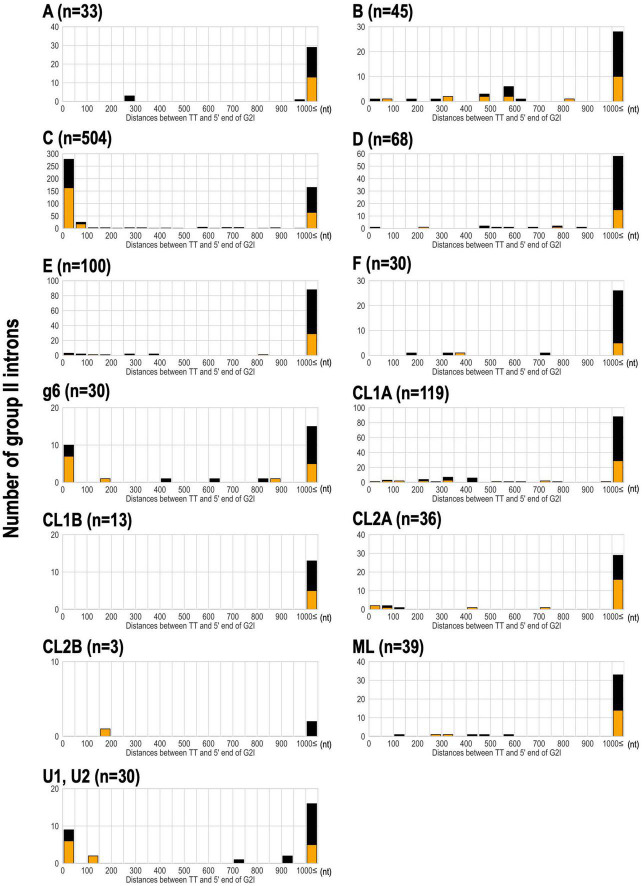
Analysis of the distance between the transcriptional terminator and the 5′ end of G2Is for each IEP type in bacteria. The distance between the transcriptional terminator and the 5′ end of the G2I was calculated, and the number of G2Is at each distance is represented as a histogram for each IEP type. L-shaped terminators are shown in orange boxes and I-shaped terminators are shown in black boxes. G2Is with bacterial-g1 IEPs are not shown because their 5′ ends were not identified in this analysis. TT: transcriptional terminator.

We have shown that bacterial-C-type G2Is are widespread in bacteria and are increasing in many species. This is because bacterial-C-type G2Is are inserted immediately after rho-independent transcription terminators during transposition (Robart et al., 2007). Therefore, their effect on the host gene(s) is limited because these G2Is do not break the coding sequences in the host genome (Robart et al., 2007). Therefore, there are very few opportunities for bacterial-C-type G2Is to be transcribed (Robart et al., 2007; Mohr et al., 2018). It is well documented that transcription terminators in the genome and bacterial C-type G2Is are in close proximity (Dai and Zimmerly, 2002; Waldern et al., 2020), but there is still limited knowledge of other IEP types. Therefore, we comprehensively analyzed the positional relationships between the G2Is identified in this study and the previously predicted rho-independent transcription terminators (Mitra et al., 2011). For 304 bacterial genomes that are included in the transcription terminator database and have G2Is, the distance from the 5′ end of G2I to the transcription termination site was calculated (Figure 5). Our results showed that bacterial-C-type G2Is are dominated by sequences inserted immediately after the transcription terminator. As in the case of the bacterial-C-type G2Is, nearly half the G2Is that occurred immediately after the transcription terminator contained other common IEP types, such as bacterial-g6 and unclassified U1 and U2. The bacterial-C, g6, U1, and U2 types of IEP were located close to each other on the IEP phylogenetic tree (Figure 2A). The distribution of these IEPs on the phylogenetic tree suggested that the functions of these different IEP types are close to those of the bacterial C-type IEPs. By contrast, other IEP-type G2Is tended to be located more than 1,000 bases from the transcription termination sites. We thought that these G2Is can enter the coding sequences of gene and cause disruption, or occur in intergenic regions, without a rho-independent transcription terminator. We next examined how the type of transcription terminator contributes to the increase in G2Is for each IEP type. There are two major types of rho-independent transcription terminators, L-shaped and I-shaped. The L-shaped transcription terminator is defined as the presence of four or more U residues in the tail region immediately after the stem–loop structure of the 3′ untranslated region (UTR) of the transcript. All of the other transcription terminators are classified as I-shaped (Mitra et al., 2011). No clear tendency was observed for any IEP type to insert preferentially into the L-shaped or the I-shaped transcription termination regions (Figure 5).

Rho-independent transcription terminators exist across a wide range of phyla, and in fact bacterial-C-type G2Is are increasing in many species. So, why does Firmicutes have a significant number of bacterial-C-type G2I? One explanation is that Firmicutes has a higher ratio of rho-independent transcription terminators to the total number of genes than other phyla, and a higher ratio of genes on the leading strand to the total number of genes (Mitra et al., 2011; Mao et al., 2012). To further examine the explosive increase in bacterial-C-type G2Is in Firmicutes, we considered the role of rho-independent transcription terminators in that phylum. In *Escherichia coli* (phylum Proteobacteria), the transcription and translation processes are coupled, so the RNA polymerase and ribosome are usually linked and move at the same rate. It has been suggested that the ribosome linked to the RNA polymerase inhibits the formation of RNA hairpins, resulting in partial suppression of the majority of rho-independent transcription terminators (Wang et al., 2019). When the RNA polymerase and the ribosome are separated from each other as the movement of the ribosome slows, transcription termination by rho is promoted (Artsimovitch, 2018). Therefore, rho is thought to be responsible for a wide range of transcription termination phenomena in *E. coli*. However, transcription termination in *Bacillus subtilis* (phylum Firmicutes) is less dependent on rho, and rho-independent termination is the main transcription termination mechanism (Johnson et al., 2020). This characteristic transcription termination control in *B. subtilis* is considered to be common in Firmicutes (Johnson et al., 2020). From this evidence, we infer that transcription termination by the rho-independent transcription terminator alone is predominant in Firmicutes, and when a bacterial-C-type G2I is inserted immediately after the terminator, the G2I is rarely transcribed. This suggests that the effect of increased G2Is on the host genome is very small. It is also possible that the rho-independent transcription terminator in Firmicutes has sequence and structural features that preferentially bind to G2Is with bacterial-C-type IEPs. However, further analysis is required to clarify this. Incidentally, it seems that CL-type G2Is, which are abundant in Cyanobacteriota, are inserted in positions unrelated to the terminator. If so, why do the CL-type G2Is of Cyanobacteriota increase? Therefore, in addition to local sequence features, such as terminators, we decided to analyze the relationships between genome-level structures and G2I insertions.

### Bacterial Genomic Structure and Integration of Group II Introns Into the Genome

It has been shown that bacterial-C-type G2Is are preferentially inserted into the template strand for lagging-strand synthesis, which exposes more single-stranded DNA regions during host DNA replication (Robart et al., 2007). Strand-specific insertion has been reported even when there is no insertion downstream from the transcription terminator in the genome. A well-studied G2I, Ll.LtrB, with an ML-type IEP has been shown to be strand-specifically inserted into replication forks in the retrotransposition pathway (Ichiyanagi et al., 2002; Garcia-Rodriguez et al., 2019). Therefore, to examine the possibility that each IEP type is inserted into a specific DNA strand, we investigated whether G2Is occur on the leading strand or lagging strand in genomes for which information on the origin of DNA replication (*ori*) was available. The insertion bias (IB) score was calculated as the ratio of the number of G2Is on the lagging strand to the number of G2Is on the leading strand, and the bias of the insertion strand was quantified. In this way, we found that the insertion strand differed for each IEP type: G2Is with bacterial-B, -C, F [g2–g5], g6, or unclassified IEP types were strongly distributed on the lagging strand (IB score ≥ 5), whereas those with bacterial-D, -E, ML, or ORF-less IEP types were moderately distributed on the lagging strand (IB score ≥ 2– < 5) (Supplementary Figure 11). Similar results were also reported by Zimmerly’s laboratory (Wu, 2018).

**FIGURE 6:**
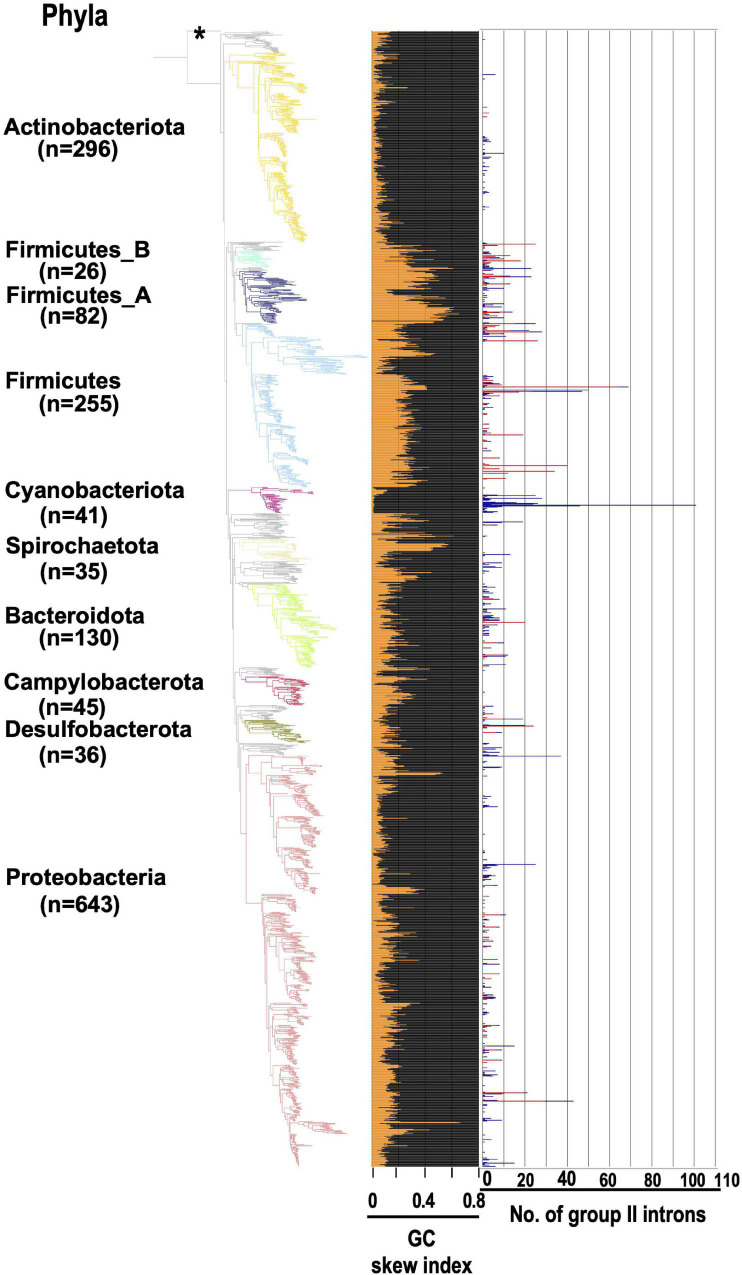
GCSI and number of G2Is in the bacterial phylogeny. The GC skew index and numbers of G2Is in representative complete bacterial genomes (1,774 genomes) are shown. Bacterial phyla are shown on left and each corresponding branch on the bacterial phylogenic tree is colored. The numbers in brackets represent the number of genomes in each phylum. The position of the outgroup [*Candidatus Saccharibacteria* oral taxon TM7x (RefSeq assembly accession: GCF_000803625.1)] is indicated by the asterisk. The orange line in the middle panel indicates the GC skew index of the longest genome in each bacterial species. The numbers of G2Is are also shown on the right (red line: G2Is with bacterial-C type IEPs; blue line: G2Is with other IEPs).

If some G2Is can distinguish each strand of the genome during their integration, what does this look like at the level of the whole genome? We conducted the following analysis using the generalized GC skew index (GCSI) (Arakawa et al., 2009), which reflects the effect of replication bias in bacterial genomes. Here, the GC skew is a measure of strand asymmetry in the distribution of guanines and cytosines. GCSI is 1.0 if the GC skew bias in each DNA sequence is strong and 0 if no bias is present. Most bacterial genomes have a GCSI of about 0.1 (Supplementary Figure 12), and genomes with a GCSI of ≤ 0.1 do not show any clear bias in GC skew (Arakawa et al., 2009). Figure 6 shows GCSI and the number of G2Is in the bacterial phylogeny. First, bacterial strains and GCSI values roughly corresponded in three groups. In Actinobacteriota and Cyanobacteriota, GCSI was low in most genomes (0.06 and 0.03 on average, respectively), whereas in Firmicutes, GCSI was relatively high (0.28 on average), although the variability was large. Moreover, as mentioned above, G2Is of the main specific IEP type were present or increased in these three taxa, such as the bacteiral-G [g1] type in Actinobacteriota, the CL type in Cyanobacteriota, and the bacterial-C type in Firmicutes (Figure 3). However, in the other phylum groups, the GCSI pattern did not appear to correspond as well to the strain, and the presence or increase of a specific IEP type was not observed.

**FIGURE 7:**
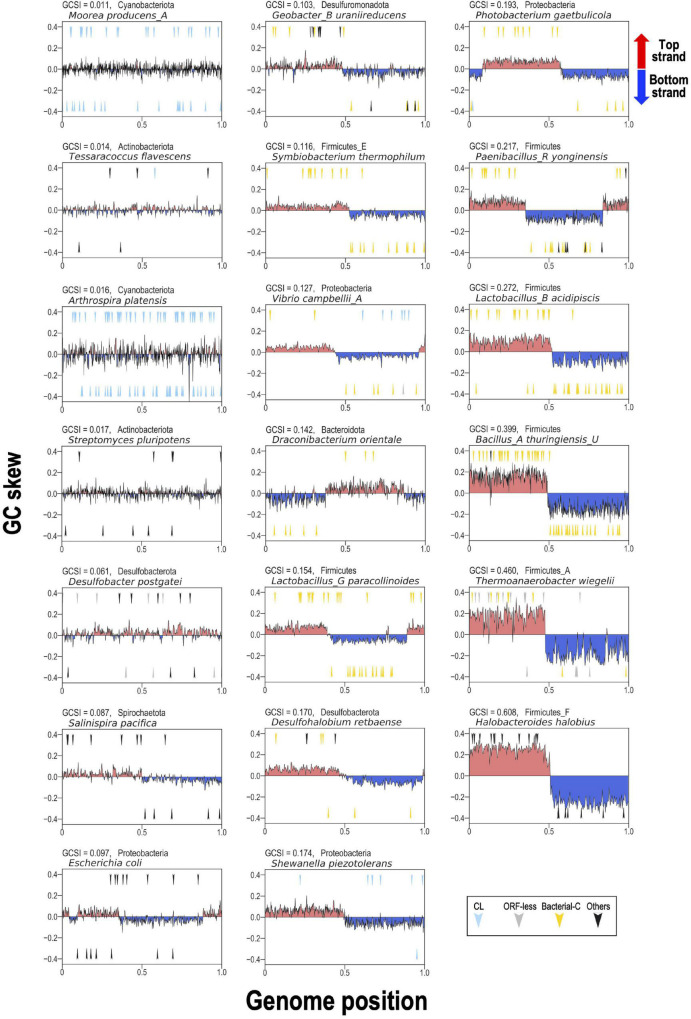
GC skew and insertion positions of G2Is in 20 representative bacterial genomes. In the boxes, the vertical axis shows the GC skew index of each genome, and the boxes are arranged in ascending order from the upper left according to the GCSI. The horizontal axis shows the relative position of each genome; the start position of the base sequence in each GenBank file is set to 0, and the end position is set to 1. The arrow in the upper half of each plot represents the insertion of G2Is into the top strand, and the arrow in the lower half represents the insertion of G2Is into the bottom strand. The colors of the arrows and the classification of G2Is are as follows: CL type (blue), ORF-less (gray), bacterial-C type (yellow), and others (black). The GCSI, bacterial phylogeny, and species name are shown in the upper left of each box. The RefSeq genome accession numbers are as follows: *Moorea producens_A*: NZ_CP017599.1; *Tessaracoccus flavescens*: NZ_CP019607.1; *Arthrospira platensis*: NC_016640.1; *Streptomyces pluripotens*: NZ_CP021080.1; *Desulfobacter postgatei*: NZ_CM001488.1; *Salinispira pacifica*: NC_023035.1; *Escherichia coli*: NC_011750.1; *Geobacter_B uraniireducens*: NC_009483.1; *Symbiobacterium thermophilum*: NC_006177.1; *Vibrio campbellii_A*: NC_009784.1; *Draconibacterium orientale*: NZ_CP007451.1; *Lactobacillus_G paracollinoides*: NZ_CP014915.1; *Desulfohalobium retbaense*: NC_013223.1; *Shewanella piezotolerans*: NC_011566.1; *Photobacterium gaetbulicola*: NZ_CP005974.1; *Paenibacillus_R yonginensis*: NZ_CP014167.1; *Lactobacillus_B acidipiscis*: NZ_LT630287.1; *Bacillus_A thuringiensis_U*: NC_022873.1; T*hermoanaerobacter wiegelii*: NC_015958.1; *Halobacteroides halobius*: NC_019978.1.

To represent these findings visually for typical bacterial examples, 20 bacterial genomes containing relatively numerous G2Is were selected. We analyzed the GC skew according to the GCSI and the insertion position of each intron in the genome (Figure 7). In genomes with high GCSI, such as the phylum Firmicutes (approximately 0.08–0.6), and a well-defined genomic structure, bacterial-C-type G2Is were inserted unevenly on one strand. In genomes with lower GCSIs (approximately 0–0.02), such as the phyla Cyanobacteriota and Actinobacteriota, G2Is other than the bacterial-C type showed no bias in the DNA strand into which they were inserted. Although these observations cannot be generalized to all bacteria, they suggest that the structural features of bacterial genomes are closely associated with the insertion of G2Is.

Recently, Watanabe reported that Cyanobacteriota species with a polyploid genome have a relatively low GCSI (Watanabe, 2020). Therefore, we looked at the relationship between the ploidy and G2I in two species with relatively low GCSI and two species with relatively high GCSI in Cyanobacteriota. In *Trichodesmium erythraeum* IMS101 (GCSI = 0.021) and *Anaebaena cylindrica* PCC 7122 (GCSI = 0.016), which have relatively low GCSIs, the former had approximately 100 chromosome copies and 24 G2Is per chromosome, and the latter had approximately 25 chromosome copies and 5 G2Is per chromosome. On the contrary, in the chromosomes of *Prochlorococcus marinus* str. CCMP1375 (GCSI = 0.118) and *Synechococcus* sp. WH 8103 (GCSI = 0.085), which have relatively high GCSIs, the former had 1 chromosomal copy and the latter 1–2 copies, and no G2I was present in any of the chromosomes. Considering these results, polyploidy may be involved in the increase of CL-type G2Is in Cyanobacteriota. In multicopy genomes, even if G2I is integrated into an essential gene on one chromosome, gene expression from the other chromosomes will remain intact, so G2I integration will have less effect than in haploid bacteria. Such genomic redundancy may be one of the factors supporting the growth of transposable elements, such as G2Is.

Finally, all the systematic G2I data analyzed in this study are summarized in Supplementary Files, which we hope will be useful for genome-scale studies of prokaryotic transposable elements in the relevant field of science.

## Materials and Methods

### Datasets

We examined 14,506 bacterial and 296 archaeal genomes in this study. The genomic sequences and their annotations were downloaded in GenBank format from the NCBI RefSeq database (O’Leary et al., 2016) in March 2019. These genomic data met both of the following conditions: (i) assembly level; complete genome or chromosome; and (ii) version status; latest. We used the Biopython module (version 1.73) (Cock et al., 2009) to read the GenBank annotation data and to calculate the GC skew. Information on the genomes used for each figure is summarized in Supplementary Table 6.

### Construction of Phylogenetic Trees for Prokaryotic Genomes

The phylogenetic trees of 27,372 bacterial and 1,569 archaeal genomes were downloaded from the Genome Taxonomy Database (version 86.2) (Parks et al., 2018) and the trees were pruned using ETE Toolkit (version 3.1.1) (Huerta-Cepas et al., 2016) to focus on specific groups of genomes in each analysis. The phylogenetic trees were visualized with iTOL (version 5) (Letunic and Bork, 2021). *Candidatus Saccharibacteria* oral taxon TM7x, a member of the CPR bacteria (RefSeq assembly accession: GCF_000803625.1), was used as the outgroup. We constructed six phylogenetic trees for: (i) representative bacteria (1,774 genomes in Figures 1, 6 and Supplementary Figure 4); (ii) representative bacteria containing G2I gene(s) (443 genomes in Figure 3); (iii) representative bacteria containing ≥ 20 G2I genes (25 genomes in Supplementary Figure 9) and all cyanobacterial genomes in the dataset (130 genomes in Supplementary Figure 10); (v) all archaeal genomes in the dataset (222 genomes in Figure 4A); and (vi) all archaeal genomes containing G2I gene(s) in the dataset (19 genomes in Figure 4C). The representative genomes are classified as either “representative genome” or “reference genome” in the RefSeq database (March 2019).

### Comprehensive Extraction of Group II Introns From Genomic Data

The pipeline used to extract G2Is from the genomic data is shown in Supplementary Figure 1A and comprised the following steps. Step 1 (extracting three groups of domains of G2I):

(i) the NCBI BLAST + (version 2.4.0) tblastn command line tool (Camacho et al., 2009) was used to search for the RT domain of the IEP. As query sequences, we used amino acid sequences of 425 RT domains in G2I IEPs (Toro and Nisa-Martinez, 2014). We set the *e*-value threshold to 1*e*-10 and extracted the sequences with query coverage > 40%. The query sequence with the highest bit score was then selected and the IEP type of this query sequence was defined as the IEP type of the subject sequence.

(ii) To search for RNA domains V and VI of the G2I, the Infernal (version 1.1.2) program cmsearch was used (bit score threshold: > 24, -nohmm option) (Nawrocki and Eddy, 2013). Here, we used the RNA secondary structure model Intron_gpII (ID: RF00029) registered in the Rfam database^1^ (Kalvari et al., 2018).

(iii) To detect ORF-less-type G2Is, we also searched for the RNA domains I–IV of the G2Is with both the BLAST + (version 2.4.0) blastn command line tool (*e*-value threshold: 1*e*-10) and the Infernal (version 1.1.2) program cmsearch (*e*-value threshold: 1*e*-2, -rfam option). For the blastn search, 337 G2I sequences in prokaryotes registered in the Database for Bacterial Group II Introns^2^ (Candales et al., 2012) were manually selected as query sequences, and the sequences with query coverage > 60% were extracted. For the cmsearch search, we used RNA secondary structure models (IDs: RF01998, RF01999, RF02001, RF02003, RF02004, RF02005, and RF02012) registered in the Rfam database.

The following two steps were used to judge whether each detected domain was included in a single G2I. Step 2: When the distance between the regions with similarity to RT and domain V was ≤ 1,300 bases (Supplementary Figure 1B), we considered that the RT domain and domain V were in the same G2I. Step 3: When the distance between either domain I, II, III, or IV and domain V was ≤ 1,300 bases (Supplementary Figure 1C), we considered that either domain I, II, III, or IV and domain V were in the same G2I. The resulting G2I dataset was checked manually and corrected where necessary (Supplementary Table 2B and Figure 4).

### Prediction of Intron-Encoded Protein Sequences

From our preliminary survey, it was apparent that there are non-canonical IEP sequences, such as those whose ORFs are interrupted by stop codons (Supplementary Figure 3). Therefore, in this study, the entire IEPs (or the partial sequences of IEPs) were extracted from the peripheral sequences of the identified RT domains and classified as canonical IEP or non-canonical IEP sequences. After 12,153 RT domain sequences were extracted in the previous section (“Comprehensive Extraction of G2Is from Genomic Data”), we obtained the nucleotide sequences corresponding to the 1,000 bases upstream from the RT domain to the 200 bases downstream from domain VI in each genomic sequence (Supplementary Figure 1A, Step 1). We then searched for the nucleotide sequences corresponding to IEP amino acid sequences around these RT domains with the tblastn command (Camacho et al., 2009). Each subject sequence with the highest bit score was selected, and a total of 12,153 IEP amino acid sequences were obtained. Here, the amino acid sequences of each IEP of the 318 “Eubacterial” G2Is registered in Zbase was used as queries (Candales et al., 2012). We selected 1,065 sequences in which a stop codon appeared other than at the end of the subject sequence, and called these sequences “interrupted sequences.” To reduce the number from these 1,065 sequences before the construction of a phylogenetic tree, clustering was performed with CD-HIT (version 4.8.1) (Fu et al., 2012) with a threshold of 85% sequence similarity, and 352 clusters were extracted. By selecting one representative sequence from each cluster, a representative sample of 352 “interrupted sequences” was obtained.

For the 11,088 IEP sequences that were not “interrupted sequences,” the ORFs and the conserved domains in each IEP were predicted, and if the conserved functional domain was missing, it was considered a non-canonical IEP. That is, the ORF was predicted with ORFfinder^3^ around each RT domain in these 11,088 nucleotide sequences. This process was the source of the 11,088 IEP sequences. Because there are many frames in an ORF that differ from that of the IEP, the IEP sequences in these ORFs were selected with the blastp command, and 11,088 IEP ORFs were obtained. Next, because the number of “interrupted sequences” was large, it was difficult to create a phylogenetic tree and analyze the missing domains, so clustering was performed again using CD-HIT (version 4.8.1) with a threshold of 85% sequence similarity. This yielded 1,597 clusters. Using MEME Suite (Bailey et al., 2009), a total of 15 conserved sequence domains with lengths of 10–50 amino acids were set for each IEP type, and those missing about 5 or more domains were manually selected as IEPs with deleted domains. Consequently, 205 ORFs with large domain deletions were obtained, and these ORFs were called “short ORFs.” Finally, 557 ORFs (the sum of “interrupted sequences” and “short ORFs”) were designated “non-canonical IEPs,” and the remaining 1,392 ORFs were designated “canonical IEPs.”

### Construction of Phylogenetic Tree of Intron-Encoded Proteins

The dataset used contained 1,949 IEP sequences, including 1,392 sequences of canonical IEPs and 557 sequences of non-canonical IEPs (see “Prediction of IEP Sequences”). If the RT domain sequence of a non-canonical IEP was interrupted by a stop codon, the relevant stop codon was excluded. To construct a phylogenetic tree of the IEPs, MAFFT E-INS-i (version 7.310) (Katoh and Standley, 2014) was used to prepare a multiple alignment of the 1,949 IEP sequences, which was trimmed with trimAl (version 1.2, gappyout option) (Capella-Gutierrez et al., 2009). The phylogenetic tree was constructed with the maximum likelihood method with RAxML (version 8.2.10) (Stamatakis, 2014) using the LG + Γ model and 100 bootstrap replicates (raxmlHPC-PTHREADS-SSE3 -f a -N 100 -m PROTGAMMALG). The phylogenetic tree was visualized with iTOL (version 5).

### Calculation of Distances From Group II Intron Insertion Sites to Transcription Termination Sites

To determine which IEP-type G2Is are inserted immediately after the rho-independent transcription terminator, the distances between the rho-independent transcription terminators and the 5′ ends of the G2Is in the bacterial genomes were calculated. First, prediction data for rho-independent transcription terminators that included information on the type of terminator (L-shaped or I-shaped) were downloaded from the WebGeSTer DB (last updated: June 06, 2012) (Mitra et al., 2011), and used as “the best or strongest candidate terminators.” Because the 5′ end of a G2I is expected to be located 5′ upstream from the start codon of an IEP and within about 300–1,100 bases of it (Supplementary Figure 1), we searched for the 5′ end sequence within the region within 1,200 bases upstream from the IEP. Because files of the 5′ end consensus sequence of G2Is have already been published for each of the nine IEP types (bacterial-A, -B, -C, -D, -E, -F [g2–g5], ML, CL1, and CL2) (Waldern et al., 2020), we searched for regions with similarity to these consensus sequences within the 1,200 bases upstream from the IEPs. The positions of the 5′ ends were then mapped onto the genome of each bacterium with the following method. Using the hmmbuild and hmmpress commands of HMMER (version 3.1b2), a hidden Markov model (HMM) profile was created for each IEP type from multiple-alignment-containing files of consensus sequences (Mistry et al., 2013; Wheeler and Eddy, 2013). The nhmmscan command was then used to identify the 5′ end. For this, the default values were used for the parameters, and only hits with a bit score of ≥ 10 were selected. If there were multiple 5′ end candidates, the position with the highest bit score was selected. There are no data on the 5′-end consensus sequence of the bacterial-G [g1]-type G2Is, and the 5′-end sequence could not be determined, even when the similarity of these G2Is to other IEP types was used, so bacterial-G [g1]-type G2Is were excluded from the present study. Finally, the distance from the 5′ end of the G2I to the 3′ end of the rho-independent transcription terminator was calculated.

### Calculation of Insertion Bias Score

To quantify the bias in the DNA strand into which a G2I is inserted when it is integrated into the genome, we first attempted to identify each strand based on the origin of DNA replication (*ori*) and the terminus of DNA replication (*ter*). Predictive data for bacterial DNA replication origins were obtained from the DoriC database (version 10.0) (Luo and Gao, 2019). Positions separated from *ori* by half the chromosome length were defined as *ter*. Among 447 representative bacterial genomes with G2Is, information on *ori* was obtained for 349 genomes, excluding bacteria not registered in DoriC. The leading and lagging strands were then distinguished by considering the positions of *ori* and *ter*. The IB scores were calculated as the ratio of the number of G2Is on the lagging strand to the number on the leading strand.

### GC Skew Analysis

The GC skew of each genome was calculated as (G – C)/(G + C), where G and C represent the numbers of guanines and cytosines, respectively, in windows of 10,000 bp, using the Biopython module (version 1.73) (Cock et al., 2009). The GC skew index, which represents the strength of the GC skew, was calculated using the G-language Genome Analysis Environment (version 1.9.1) (Arakawa et al., 2003, 2009). Information on the replicon and GCSI calculated in this study is summarized in Supplementary Table 7. To construct Figure 7, we manually selected 20 representative bacterial chromosomes containing relatively many G2Is to clarify whether differences in the GC skew index of genomes affect the genomic region into which G2Is are inserted.

### Single Regression Analysis

To calculate the correlation coefficient between the genome size and the number of G2Is, a single regression analysis was performed using scikit-learn 0.23.1 (*sklearn.linear_model.LinearRegression*) (Pedregosa et al., 2011).

## Data Availability Statement

The datasets presented in this study can be found in online repositories. The names of the repository/repositories and accession number(s) can be found in the article/Supplementary Material.

## Author Contributions

MM and AK conceived, designed the study, and wrote the manuscript. MM, SN, and ST performed the analyses and interpreted the data. MM, MT, and AK edited the manuscript. AK supervised the project. All authors have read and approved the final manuscript.

## Conflict of Interest

The authors declare that the research was conducted in the absence of any commercial or financial relationships that could be construed as a potential conflict of interest.

## Publisher’s Note

All claims expressed in this article are solely those of the authors and do not necessarily represent those of their affiliated organizations, or those of the publisher, the editors and the reviewers. Any product that may be evaluated in this article, or claim that may be made by its manufacturer, is not guaranteed or endorsed by the publisher.

## Footnotes

1
http://rfam.xfam.org/

2
http://webapps2.ucalgary.ca/~groupii/

3
https://www.ncbi.nlm.nih.gov/orffinder/
