# Supplementary material for: Distinct Expansion of Group II Introns During Evolution of Prokaryotes and Possible Factors Involved in Its Regulation
Source: Front Microbiol. 2022 Feb 28;13:849080. doi: 10.3389/fmicb.2022.849080 (PMC8919778; doi:10.3389/fmicb.2022.849080)
Supplement: Supplementary file 1 [file Presentation_1.pptx]

## Slide 1
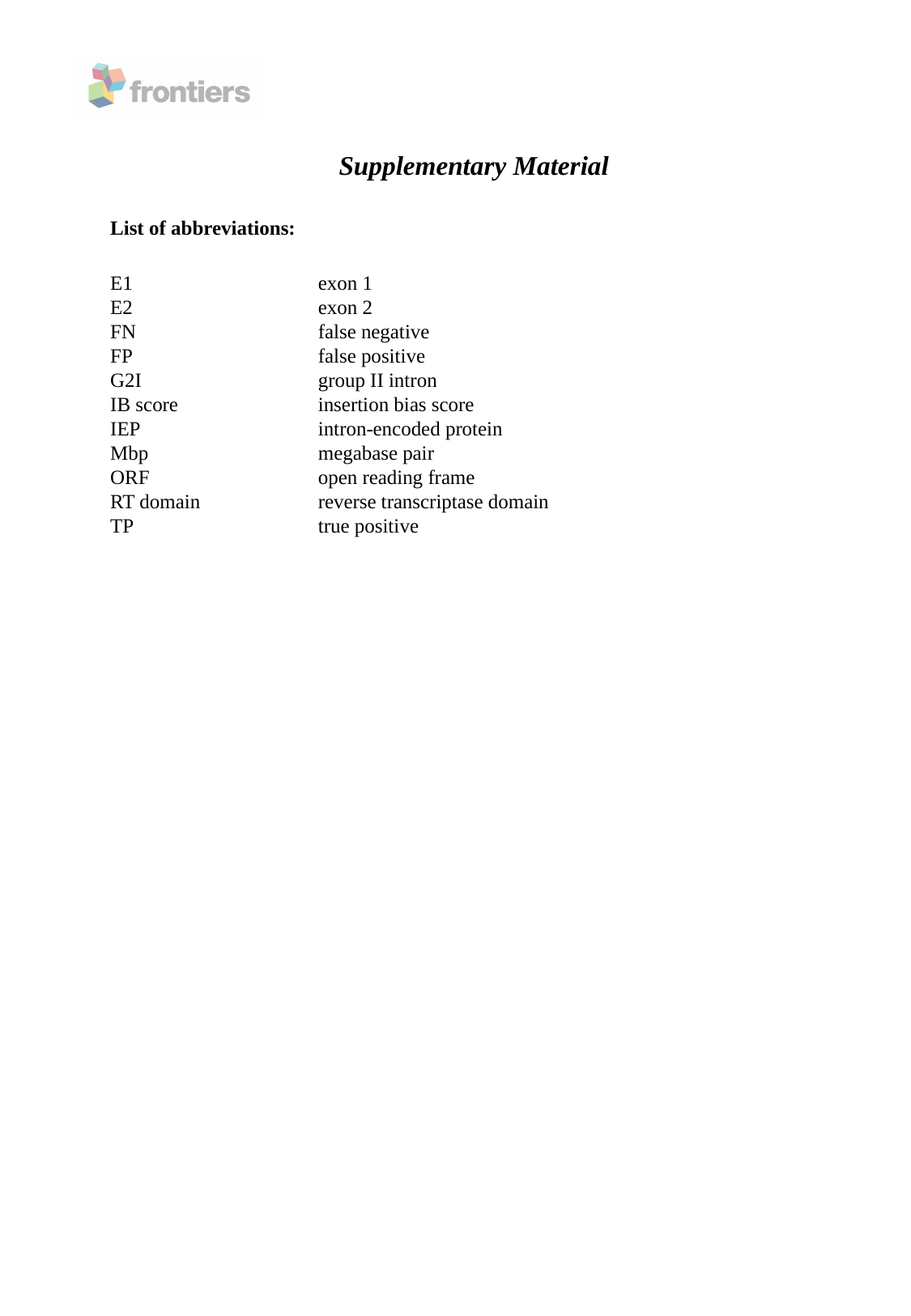

Supplementary Material
List of abbreviations:
exon 1
exon 2
false negative
false positive
group II intron
insertion bias score
intron-encoded protein
megabase pair
open reading frame
reverse transcriptase domain
true positive
E1
E2
FN
FP
G2I
IB score
IEP
Mbp
ORF
RT domain
TP

## Slide 2
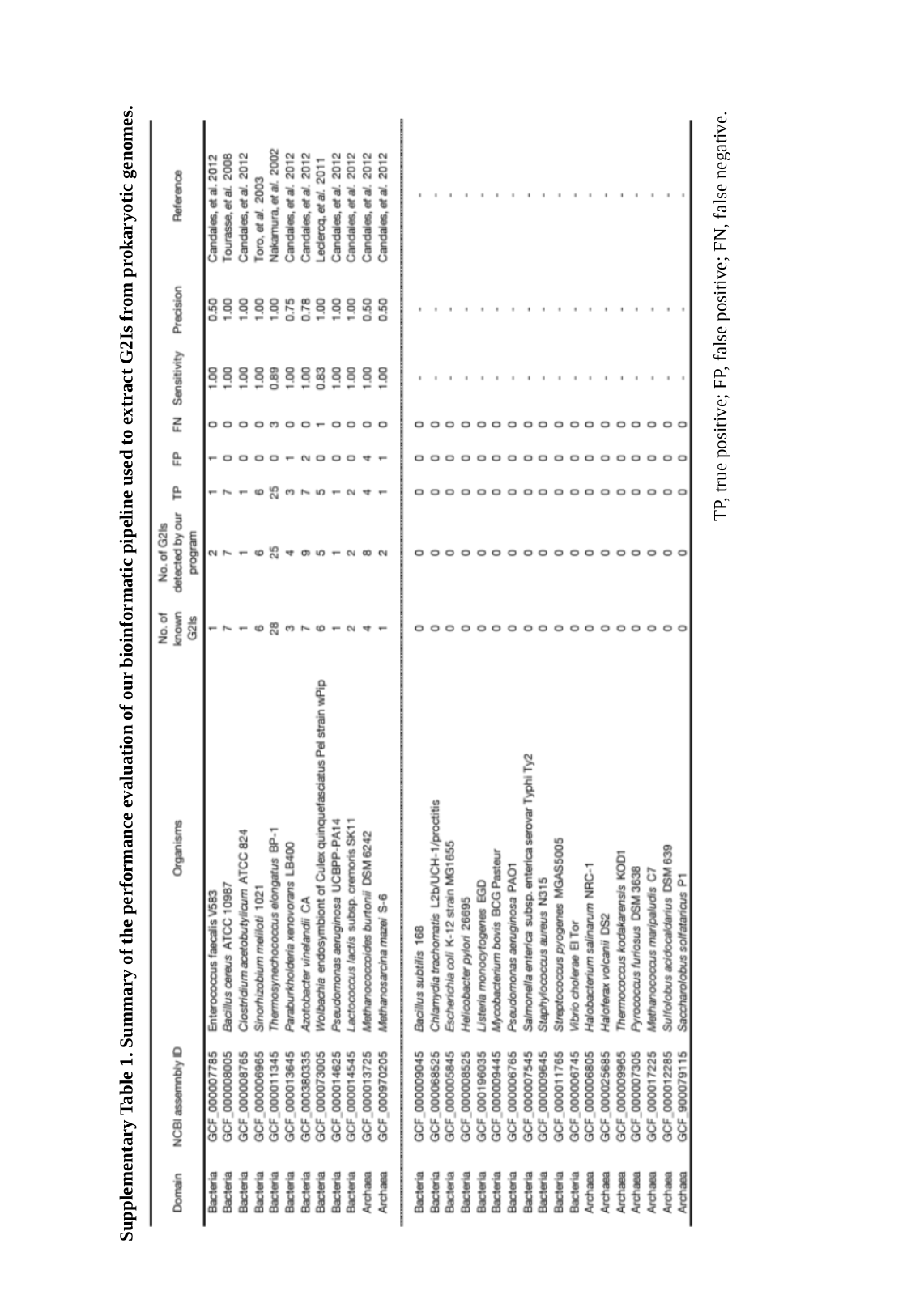

TP, true positive; FP, false positive; FN, false negative.
Supplementary Table 1. Summary of the performance evaluation of our bioinformatic pipeline used to extract G2Is from prokaryotic genomes.

## Slide 3
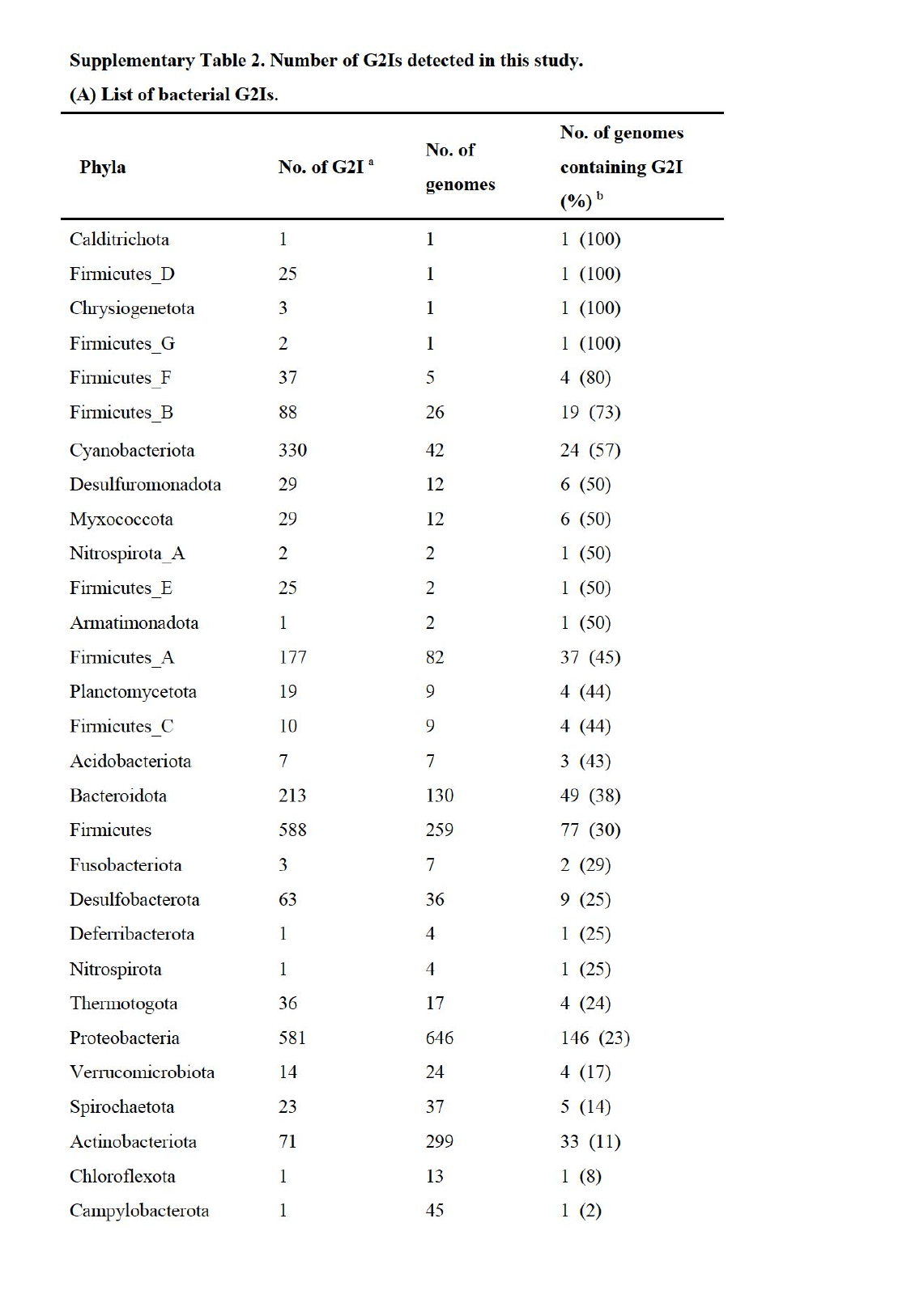

## Slide 4
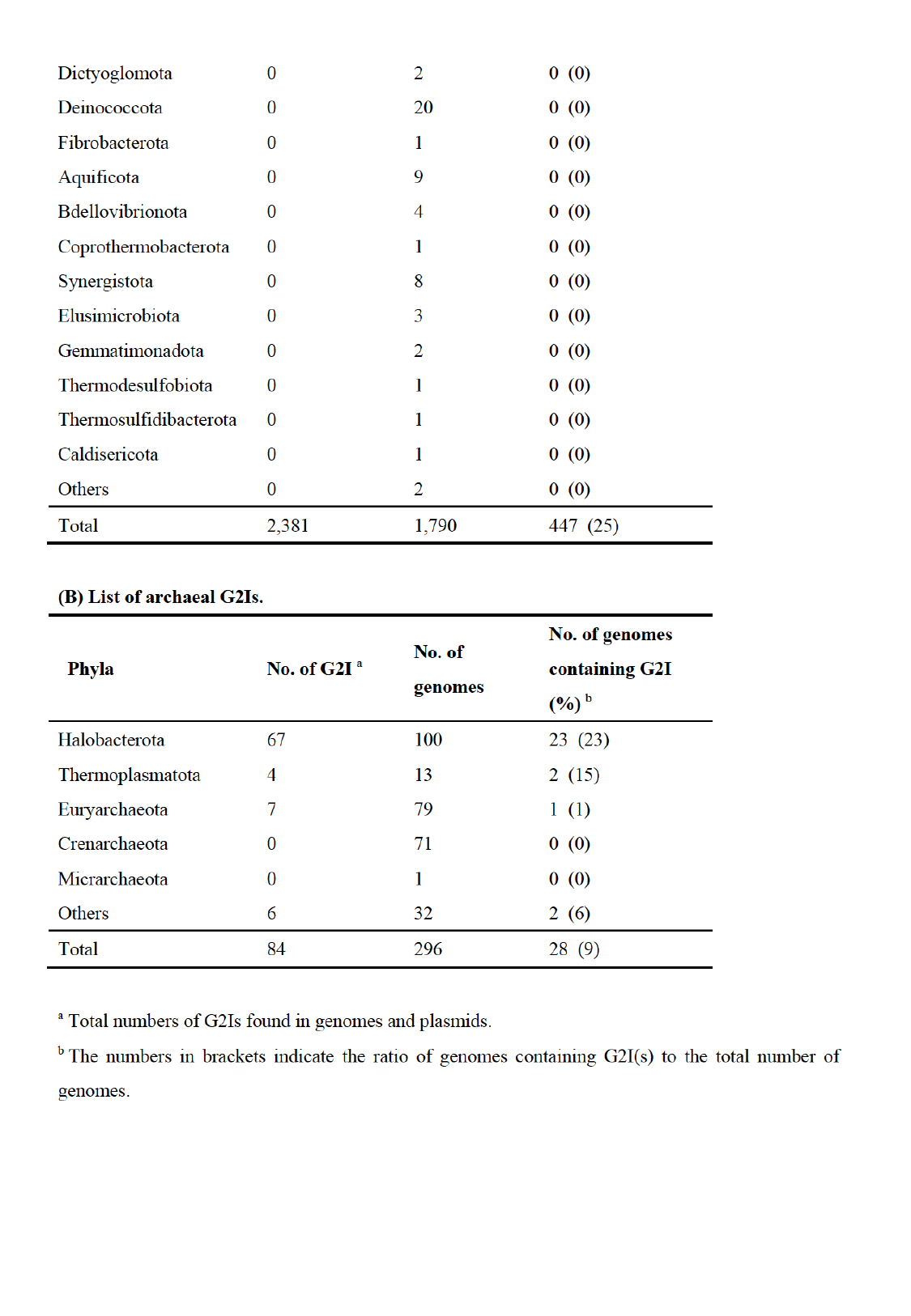

## Slide 5
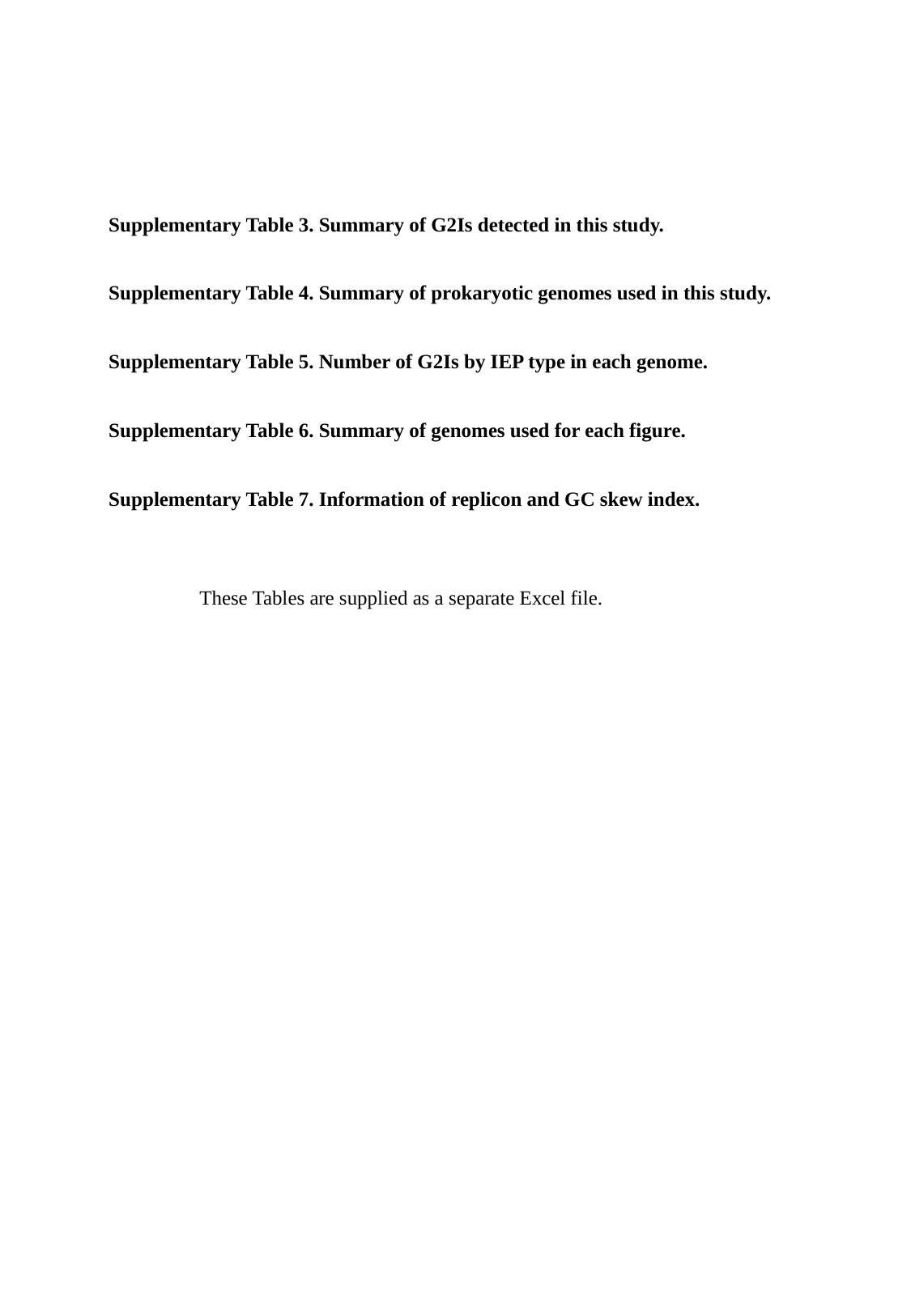

Supplementary Table 3. Summary of G2Is detected in this study.
Supplementary Table 4. Summary of prokaryotic genomes used in this study.
Supplementary Table 5. Number of G2Is by IEP type in each genome.
Supplementary Table 6. Summary of genomes used for each figure.
Supplementary Table 7. Information of replicon and GC skew index.
These Tables are supplied as a separate Excel file.

## Slide 6
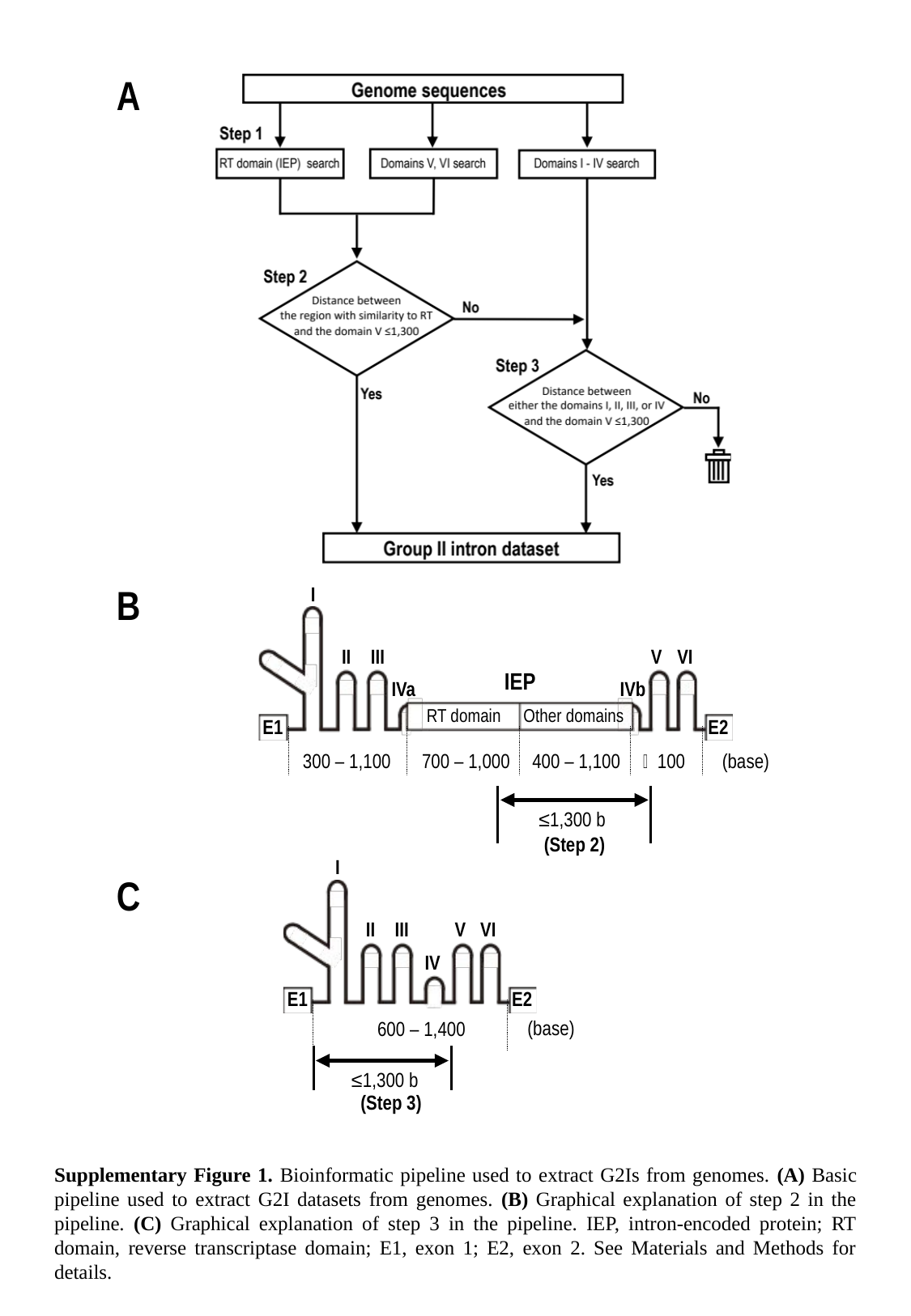

A
B
I
E1
E2
II
III
V
VI
IEP
IVa
IVb
RT domain
Other domains
(base)
300 – 1,100
700 – 1,000
400 – 1,100
〜 100
≤1,300 b
(Step 2)
I
E1
E2
II
III
V
VI
IV
C
(base)
600 – 1,400
≤1,300 b
(Step 3)
Supplementary Figure 1. Bioinformatic pipeline used to extract G2Is from genomes. (A) Basic pipeline used to extract G2I datasets from genomes. (B) Graphical explanation of step 2 in the pipeline. (C) Graphical explanation of step 3 in the pipeline. IEP, intron-encoded protein; RT domain, reverse transcriptase domain; E1, exon 1; E2, exon 2. See Materials and Methods for details.

## Slide 7
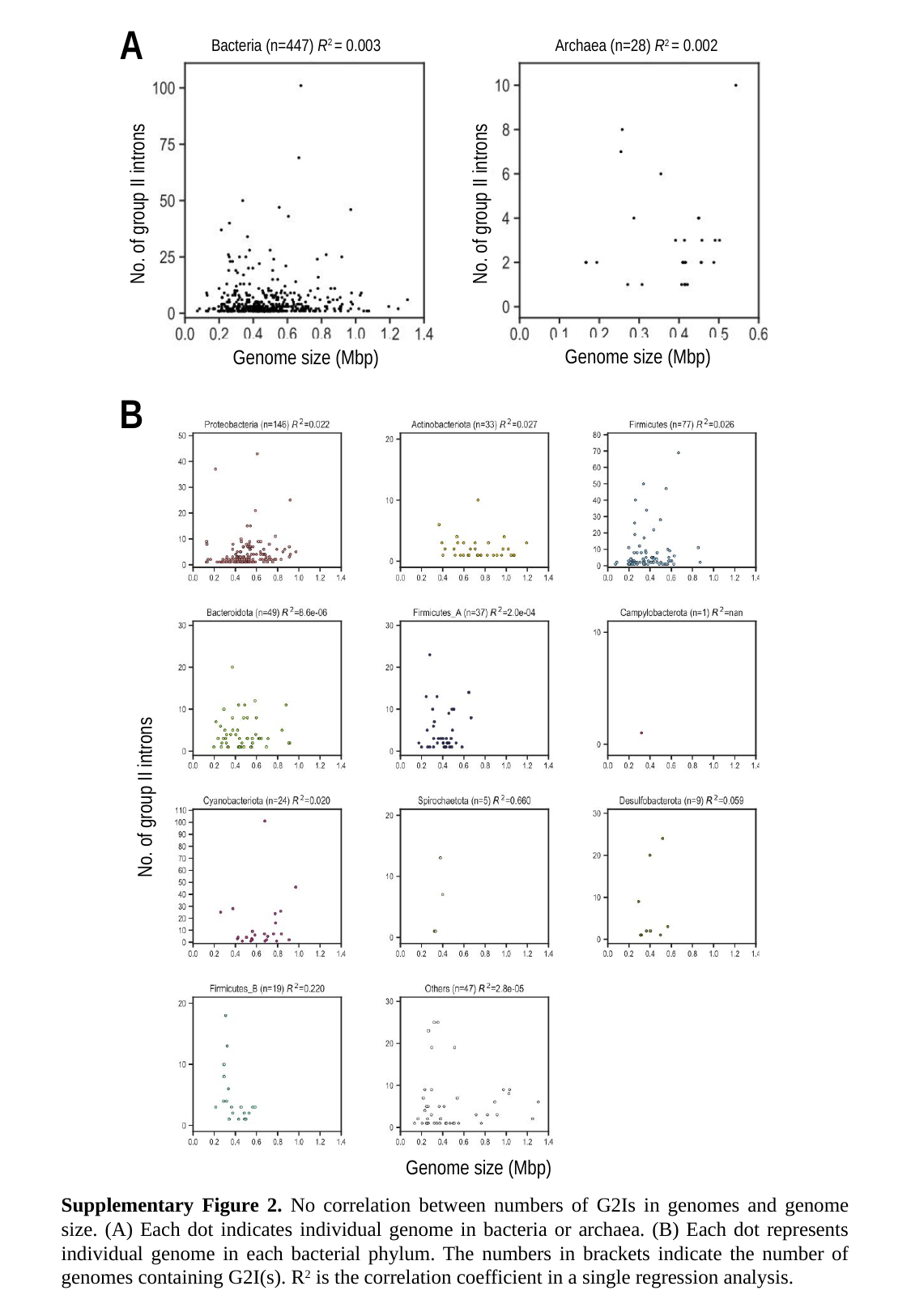

A
Bacteria (n=447) R2 = 0.003
Archaea (n=28) R2 = 0.002
No. of group II introns
No. of group II introns
Genome size (Mbp)
Genome size (Mbp)
B
No. of group II introns
Genome size (Mbp)
Supplementary Figure 2. No correlation between numbers of G2Is in genomes and genome size. (A) Each dot indicates individual genome in bacteria or archaea. (B) Each dot represents individual genome in each bacterial phylum. The numbers in brackets indicate the number of genomes containing G2I(s). R2 is the correlation coefficient in a single regression analysis.

## Slide 8
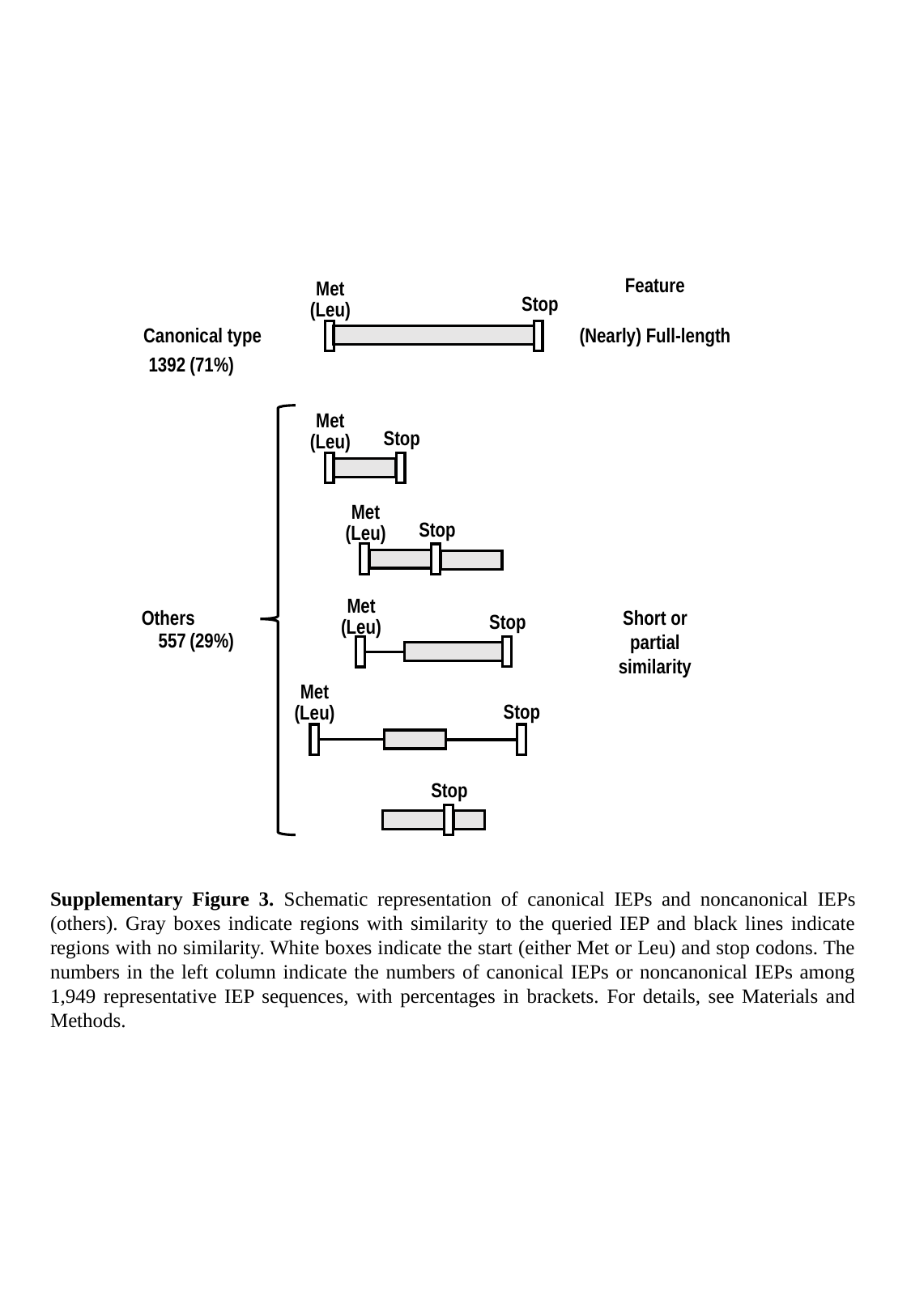

Feature
Met
(Leu)
Stop
Canonical type
(Nearly) Full-length
1392
(71%)
Met
(Leu)
Stop
Met
(Leu)
Stop
Met
(Leu)
Others
Short or partial similarity
Stop
557
(29%)
Met
(Leu)
Stop
Stop
Supplementary Figure 3. Schematic representation of canonical IEPs and noncanonical IEPs (others). Gray boxes indicate regions with similarity to the queried IEP and black lines indicate regions with no similarity. White boxes indicate the start (either Met or Leu) and stop codons. The numbers in the left column indicate the numbers of canonical IEPs or noncanonical IEPs among 1,949 representative IEP sequences, with percentages in brackets. For details, see Materials and Methods.

## Slide 9
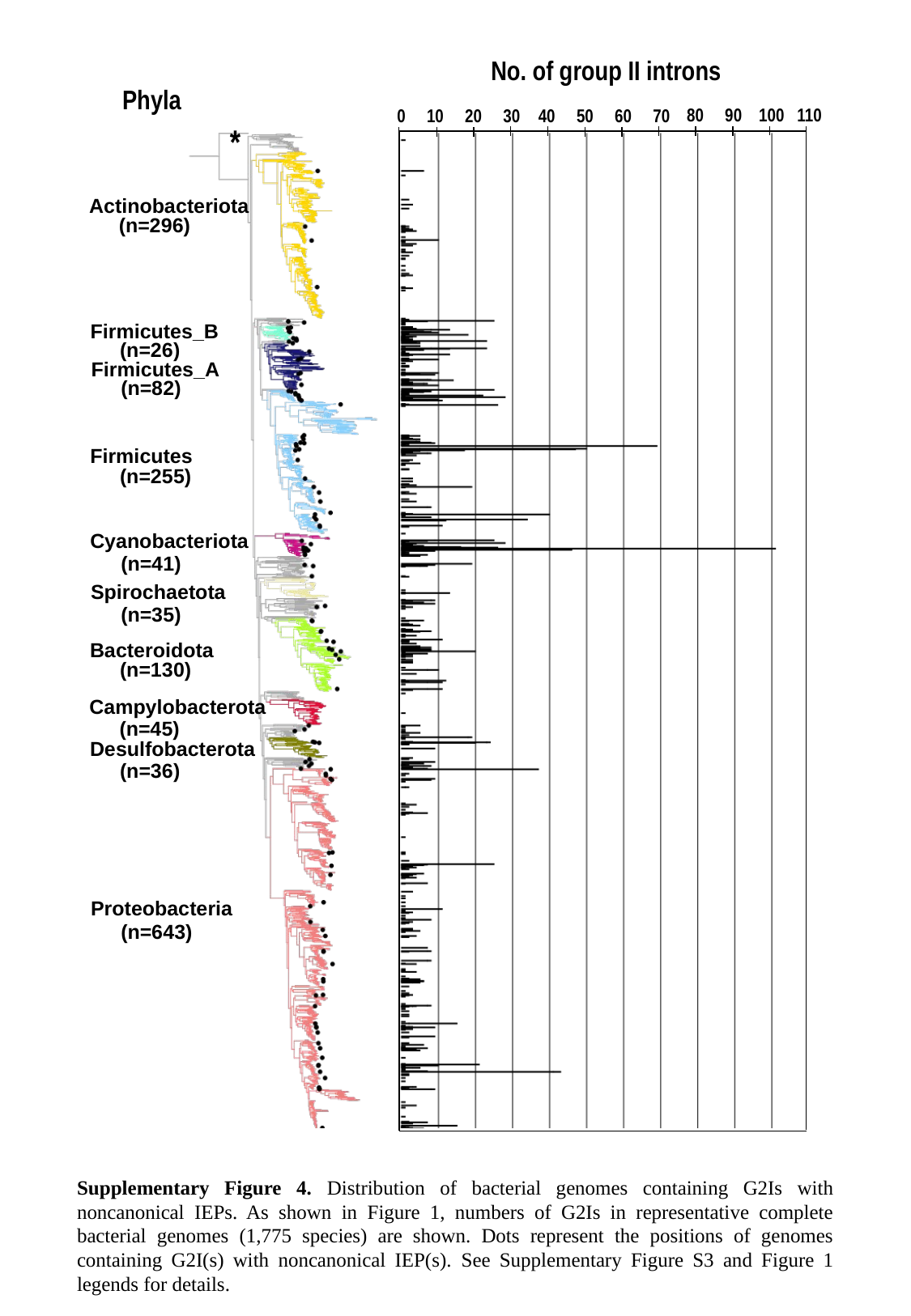

No. of group II introns
Phyla
80 90 100 110
40 50 60 70
0 10 20 30
*
Actinobacteriota
(n=296)
Firmicutes_B
(n=26)
Firmicutes_A
(n=82)
Firmicutes
(n=255)
Cyanobacteriota
(n=41)
Spirochaetota
(n=35)
Bacteroidota
(n=130)
Campylobacterota
(n=45)
Desulfobacterota
(n=36)
Proteobacteria
(n=643)
Supplementary Figure 4. Distribution of bacterial genomes containing G2Is with noncanonical IEPs. As shown in Figure 1, numbers of G2Is in representative complete bacterial genomes (1,775 species) are shown. Dots represent the positions of genomes containing G2I(s) with noncanonical IEP(s). See Supplementary Figure S3 and Figure 1 legends for details.

## Slide 10
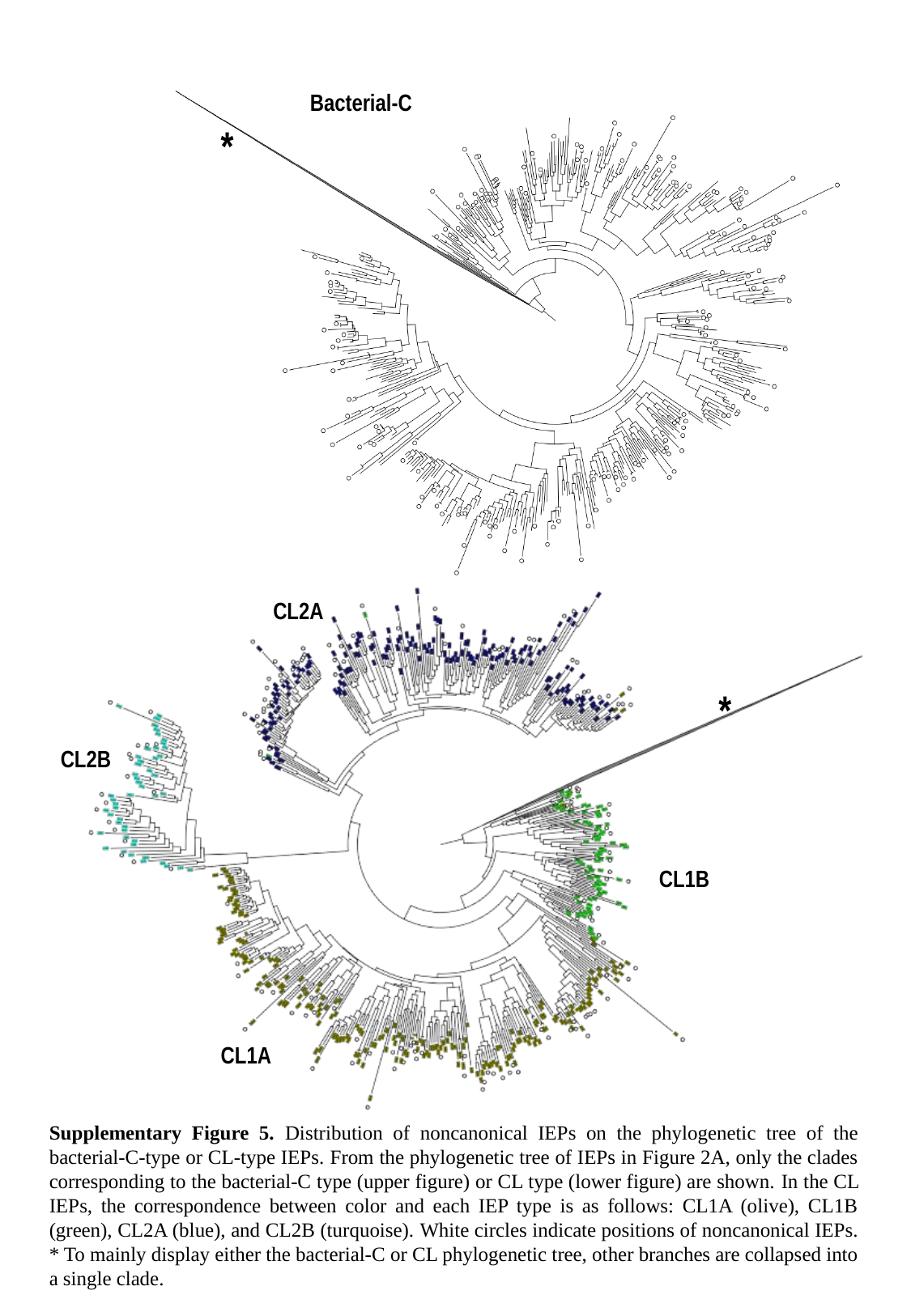

Bacterial-C
*
CL2A
*
CL2B
CL1B
CL1A
Supplementary Figure 5. Distribution of noncanonical IEPs on the phylogenetic tree of the bacterial-C-type or CL-type IEPs. From the phylogenetic tree of IEPs in Figure 2A, only the clades corresponding to the bacterial-C type (upper figure) or CL type (lower figure) are shown. In the CL IEPs, the correspondence between color and each IEP type is as follows: CL1A (olive), CL1B (green), CL2A (blue), and CL2B (turquoise). White circles indicate positions of noncanonical IEPs. * To mainly display either the bacterial-C or CL phylogenetic tree, other branches are collapsed into a single clade.

## Slide 11
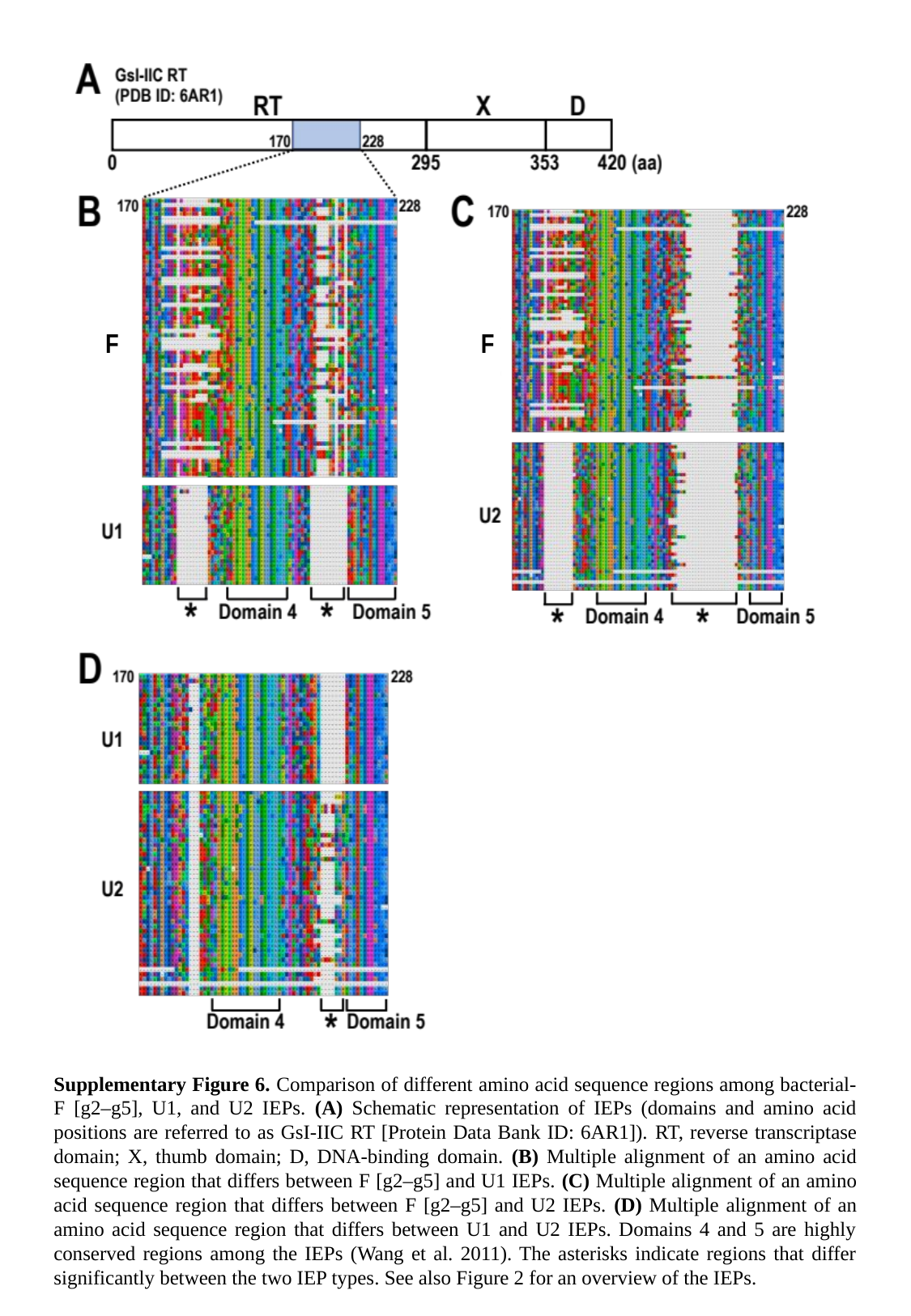

F
F
Supplementary Figure 6. Comparison of different amino acid sequence regions among bacterial-F [g2–g5], U1, and U2 IEPs. (A) Schematic representation of IEPs (domains and amino acid positions are referred to as GsI-IIC RT [Protein Data Bank ID: 6AR1]). RT, reverse transcriptase domain; X, thumb domain; D, DNA-binding domain. (B) Multiple alignment of an amino acid sequence region that differs between F [g2–g5] and U1 IEPs. (C) Multiple alignment of an amino acid sequence region that differs between F [g2–g5] and U2 IEPs. (D) Multiple alignment of an amino acid sequence region that differs between U1 and U2 IEPs. Domains 4 and 5 are highly conserved regions among the IEPs (Wang et al. 2011). The asterisks indicate regions that differ significantly between the two IEP types. See also Figure 2 for an overview of the IEPs.

## Slide 12
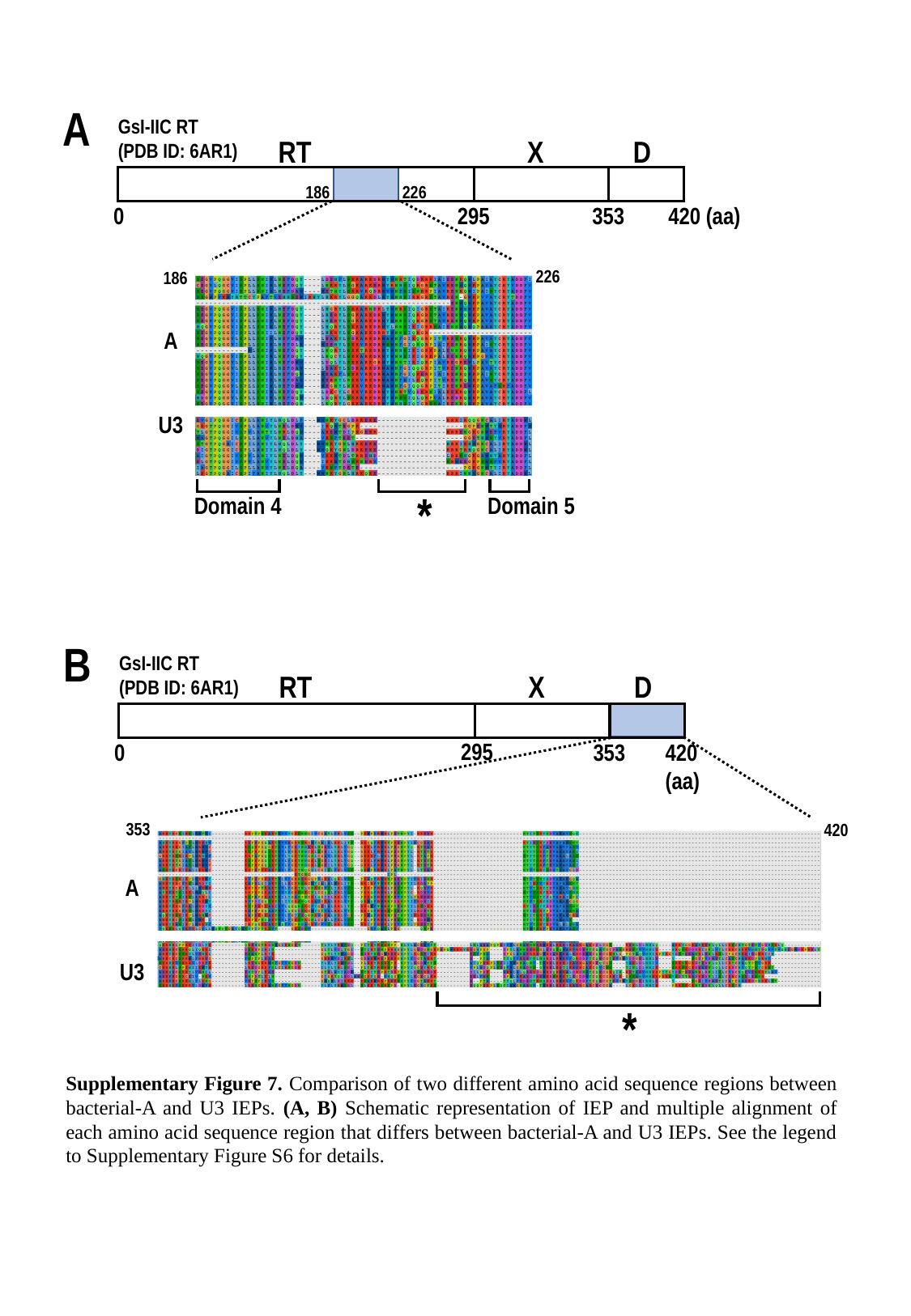

A
GsI-IIC RT
(PDB ID: 6AR1)
RT
X
D
186
226
0
295
353
420 (aa)
226
186
A
U3
*
Domain 4
Domain 5
B
GsI-IIC RT
(PDB ID: 6AR1)
RT
X
D
295
0
353
420 (aa)
353
420
A
U3
*
Supplementary Figure 7. Comparison of two different amino acid sequence regions between bacterial-A and U3 IEPs. (A, B) Schematic representation of IEP and multiple alignment of each amino acid sequence region that differs between bacterial-A and U3 IEPs. See the legend to Supplementary Figure S6 for details.

## Slide 13
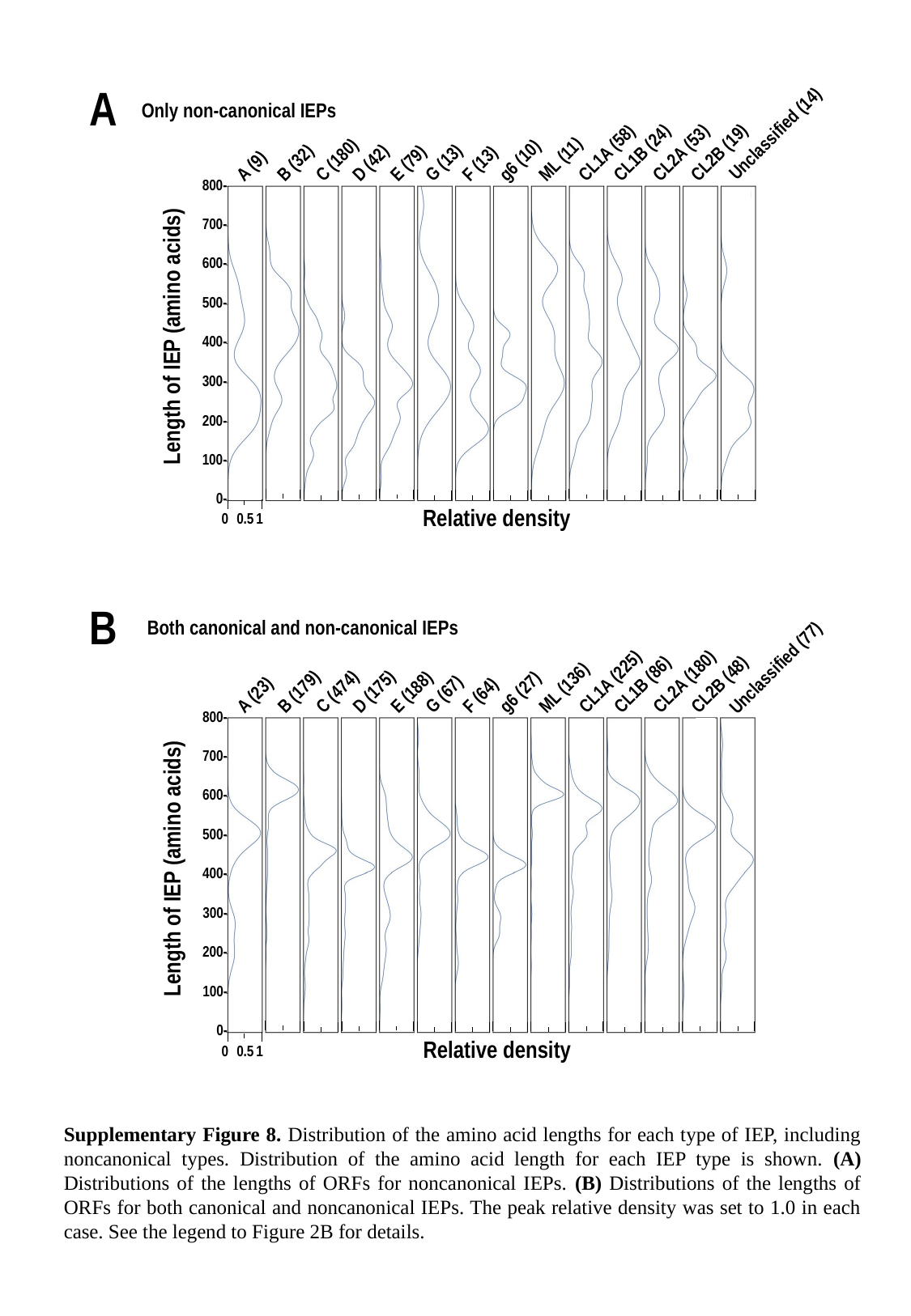

A
Only non-canonical IEPs
Unclassified (14)
CL1B (24)
CL1A (58)
CL2A (53)
F (13)
CL2B (19)
G (13)
D (42)
ML (11)
g6 (10)
E (79)
B (32)
C (180)
A (9)
800-
700-
600-
500-
Length of IEP (amino acids)
400-
300-
200-
100-
0-
Relative density
0.5
0
1
B
Both canonical and non-canonical IEPs
Unclassified (77)
CL1B (86)
CL1A (225)
CL2A (180)
F (64)
CL2B (48)
G (67)
D (175)
ML (136)
g6 (27)
E (188)
B (179)
C (474)
A (23)
800-
700-
600-
500-
Length of IEP (amino acids)
400-
300-
200-
100-
0-
Relative density
0.5
0
1
Supplementary Figure 8. Distribution of the amino acid lengths for each type of IEP, including noncanonical types. Distribution of the amino acid length for each IEP type is shown. (A) Distributions of the lengths of ORFs for noncanonical IEPs. (B) Distributions of the lengths of ORFs for both canonical and noncanonical IEPs. The peak relative density was set to 1.0 in each case. See the legend to Figure 2B for details.

## Slide 14
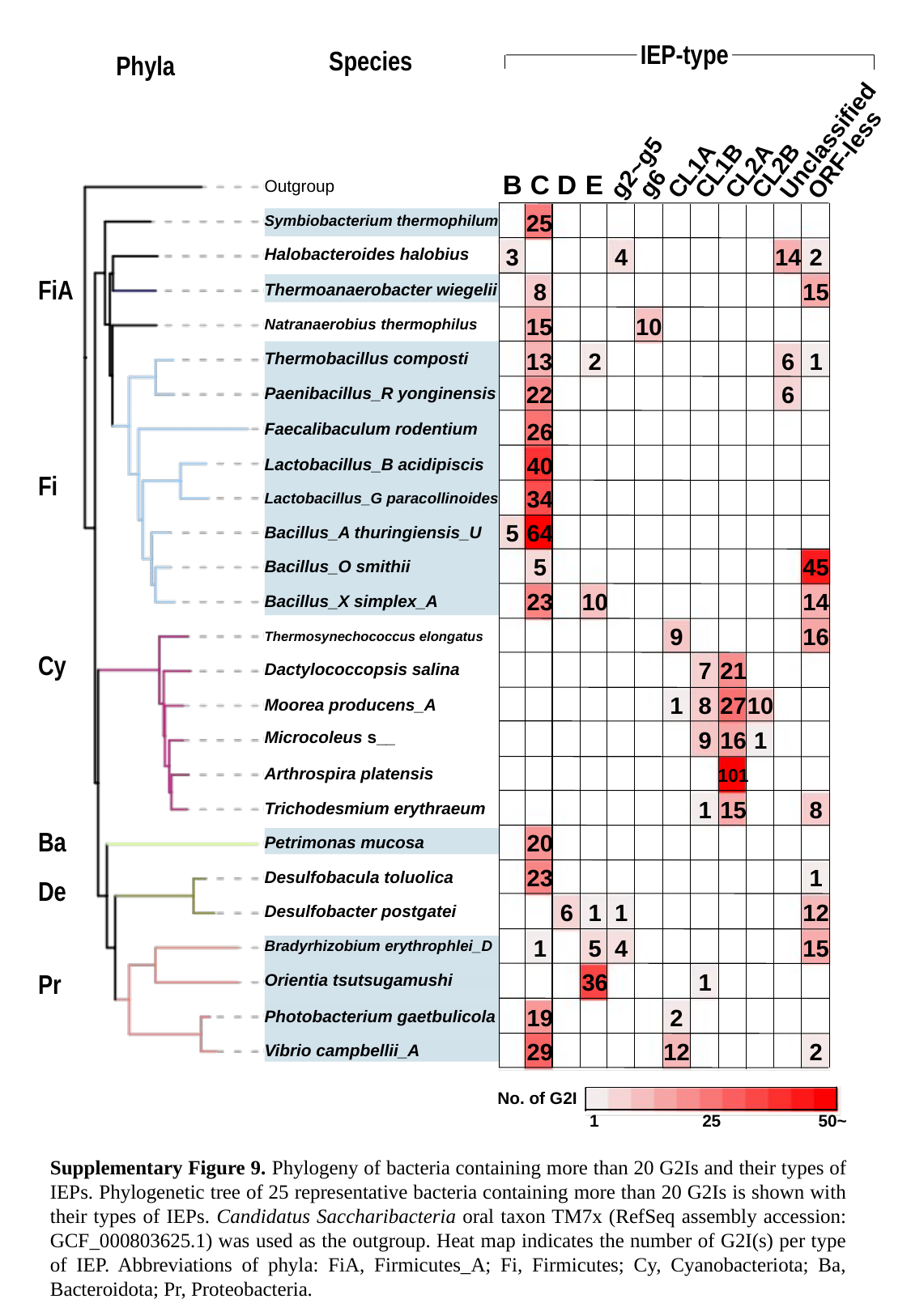

IEP-type
Species
Phyla
Outgroup
Symbiobacterium thermophilum
Halobacteroides halobius
Thermoanaerobacter wiegelii
Natranaerobius thermophilus
Thermobacillus composti
Paenibacillus_R yonginensis
Faecalibaculum rodentium
Lactobacillus_B acidipiscis
Lactobacillus_G paracollinoides
Bacillus_A thuringiensis_U
Bacillus_O smithii
Bacillus_X simplex_A
Thermosynechococcus elongatus
Dactylococcopsis salina
Moorea producens_A
Microcoleus s__
Arthrospira platensis
Trichodesmium erythraeum
Petrimonas mucosa
Desulfobacula toluolica
Desulfobacter postgatei
Bradyrhizobium erythrophlei_D
Orientia tsutsugamushi
Photobacterium gaetbulicola
Vibrio campbellii_A
Unclassified
ORF-less
g2~g5
CL1A
CL1B
CL2A
CL2B
B
C
D
E
g6
25
3
4
14
2
FiA
8
15
15
10
13
2
6
1
22
6
26
40
Fi
34
5
64
5
45
23
10
14
9
16
Cy
7
21
1
8
27
10
9
16
1
101
1
15
8
Ba
20
23
1
De
6
1
1
12
1
5
4
15
Pr
36
1
19
2
29
12
2
No. of G2I
1
25
50~
Supplementary Figure 9. Phylogeny of bacteria containing more than 20 G2Is and their types of IEPs. Phylogenetic tree of 25 representative bacteria containing more than 20 G2Is is shown with their types of IEPs. Candidatus Saccharibacteria oral taxon TM7x (RefSeq assembly accession: GCF_000803625.1) was used as the outgroup. Heat map indicates the number of G2I(s) per type of IEP. Abbreviations of phyla: FiA, Firmicutes_A; Fi, Firmicutes; Cy, Cyanobacteriota; Ba, Bacteroidota; Pr, Proteobacteria.

## Slide 15
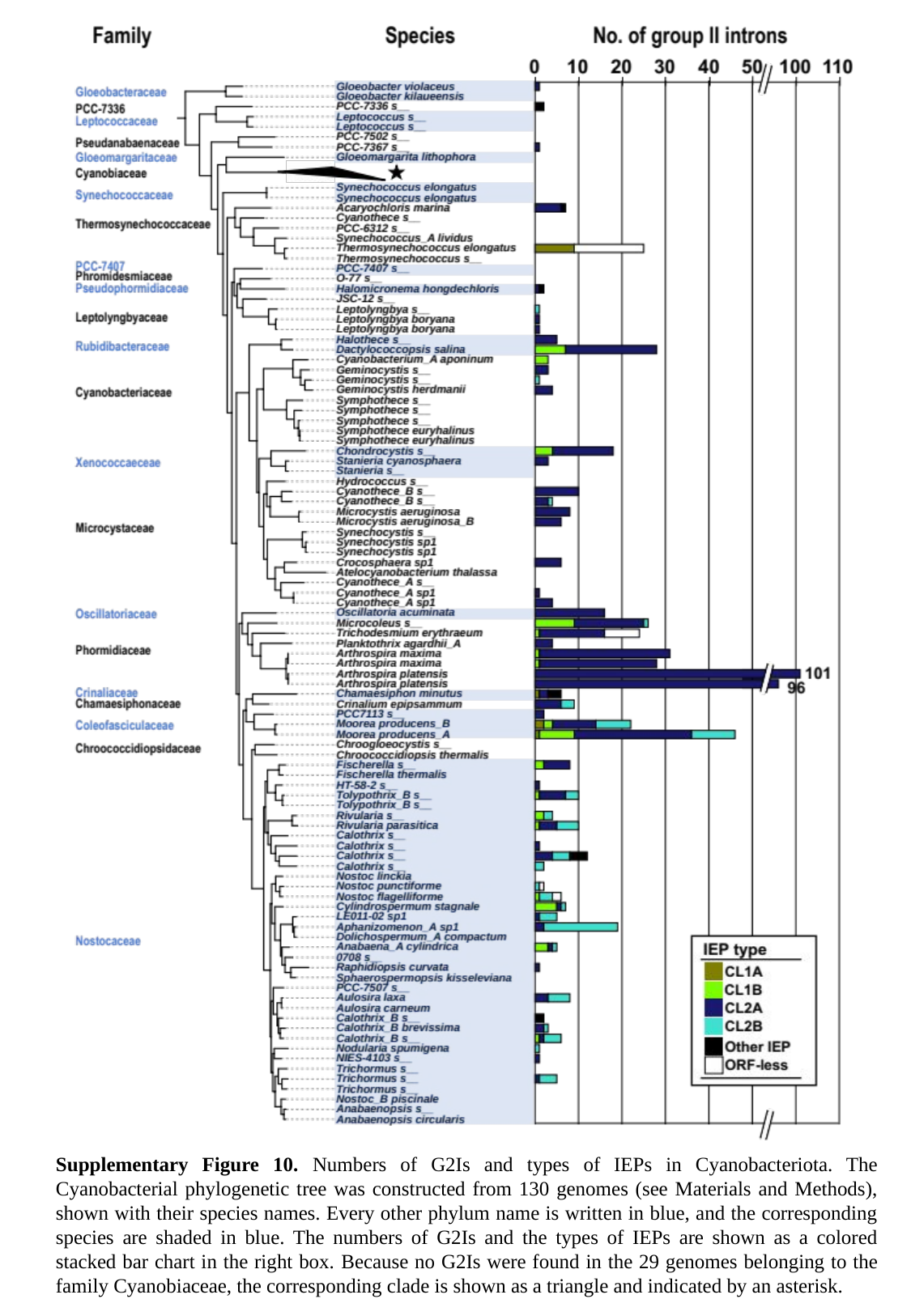

Supplementary Figure 10. Numbers of G2Is and types of IEPs in Cyanobacteriota. The Cyanobacterial phylogenetic tree was constructed from 130 genomes (see Materials and Methods), shown with their species names. Every other phylum name is written in blue, and the corresponding species are shaded in blue. The numbers of G2Is and the types of IEPs are shown as a colored stacked bar chart in the right box. Because no G2Is were found in the 29 genomes belonging to the family Cyanobiaceae, the corresponding clade is shown as a triangle and indicated by an asterisk.

## Slide 16
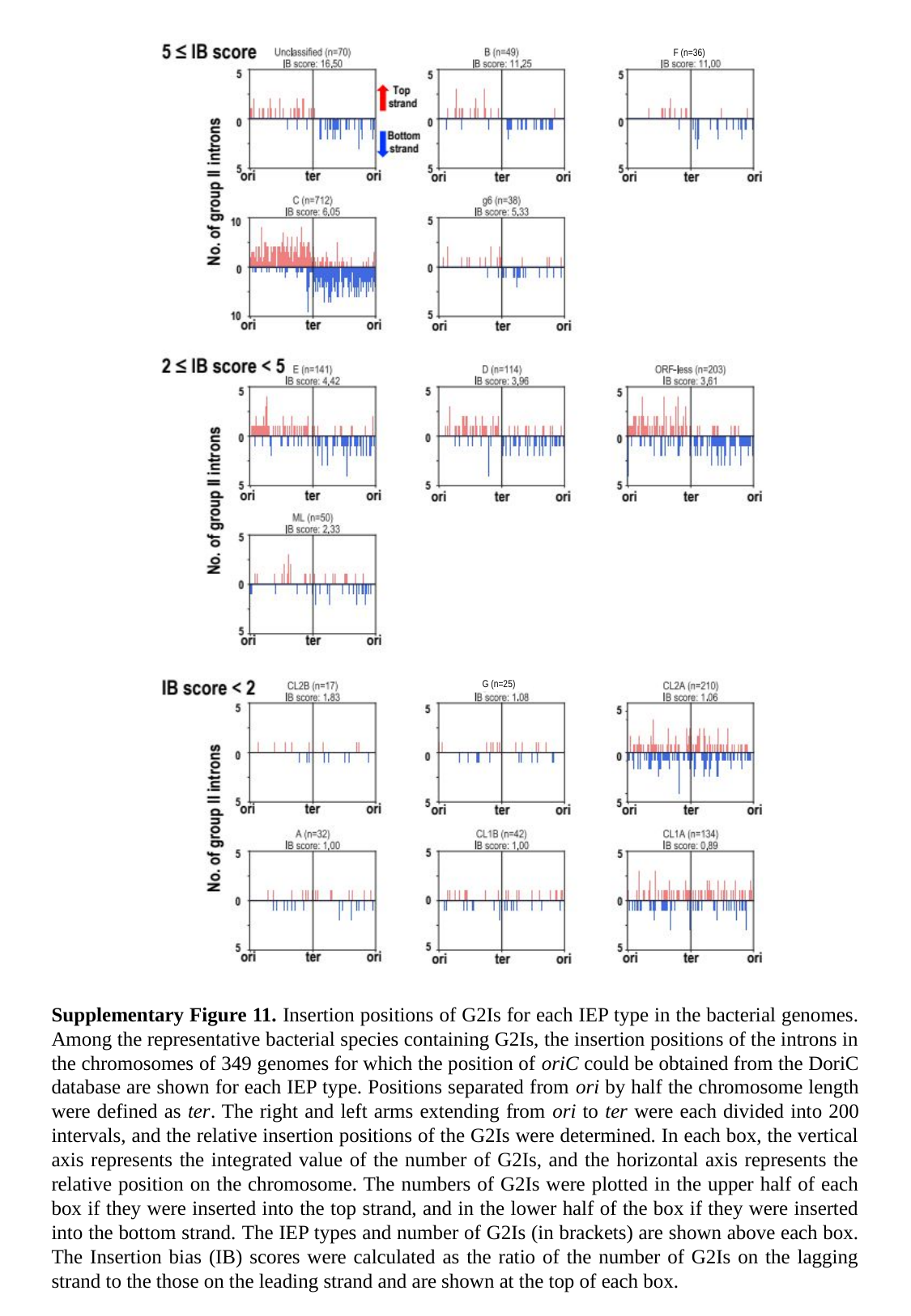

F (n=36)
G (n=25)
Supplementary Figure 11. Insertion positions of G2Is for each IEP type in the bacterial genomes. Among the representative bacterial species containing G2Is, the insertion positions of the introns in the chromosomes of 349 genomes for which the position of oriC could be obtained from the DoriC database are shown for each IEP type. Positions separated from ori by half the chromosome length were defined as ter. The right and left arms extending from ori to ter were each divided into 200 intervals, and the relative insertion positions of the G2Is were determined. In each box, the vertical axis represents the integrated value of the number of G2Is, and the horizontal axis represents the relative position on the chromosome. The numbers of G2Is were plotted in the upper half of each box if they were inserted into the top strand, and in the lower half of the box if they were inserted into the bottom strand. The IEP types and number of G2Is (in brackets) are shown above each box. The Insertion bias (IB) scores were calculated as the ratio of the number of G2Is on the lagging strand to the those on the leading strand and are shown at the top of each box.

## Slide 17
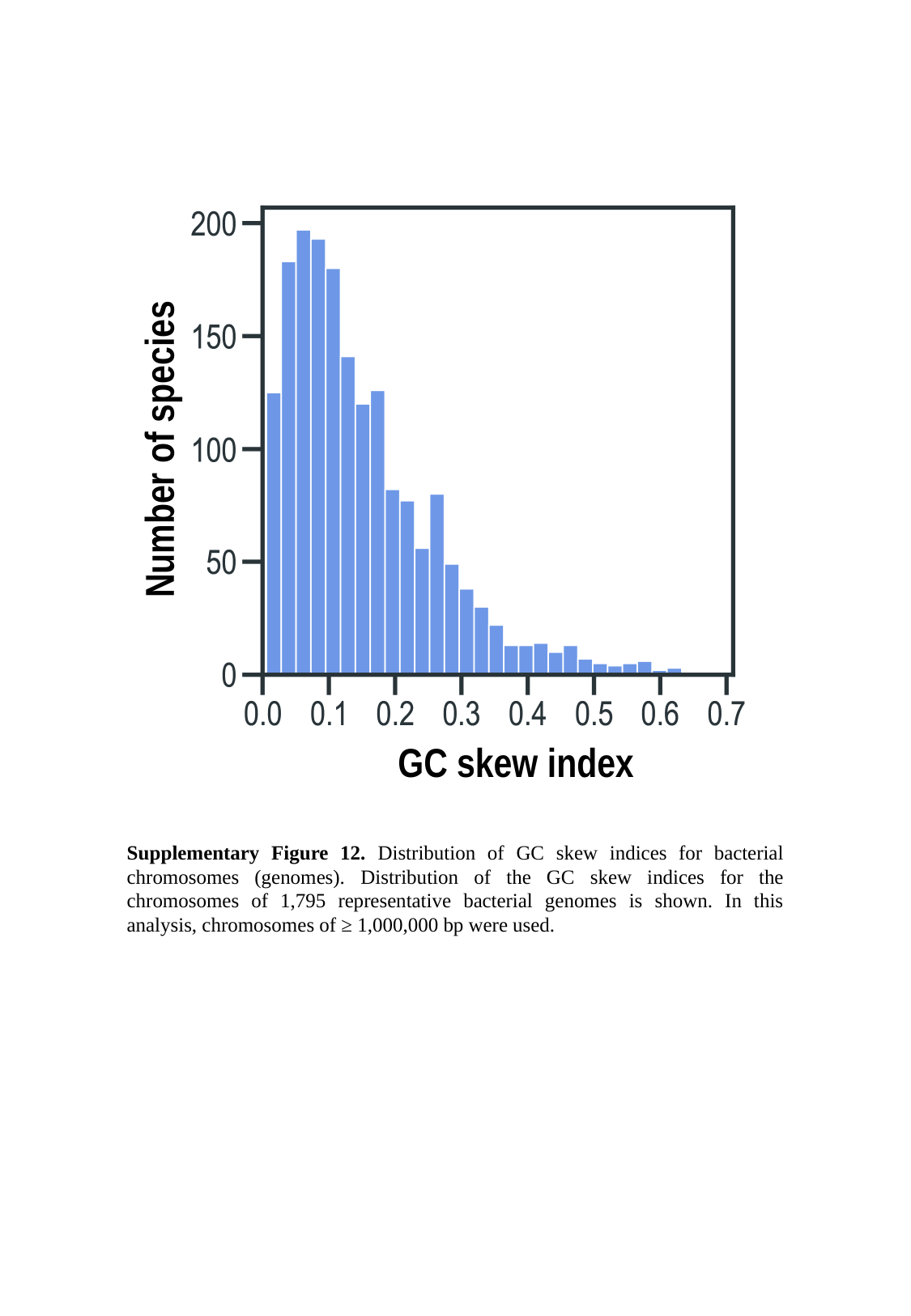

Number of species
GC skew index
Supplementary Figure 12. Distribution of GC skew indices for bacterial chromosomes (genomes). Distribution of the GC skew indices for the chromosomes of 1,795 representative bacterial genomes is shown. In this analysis, chromosomes of ≥ 1,000,000 bp were used.

## Slide 18
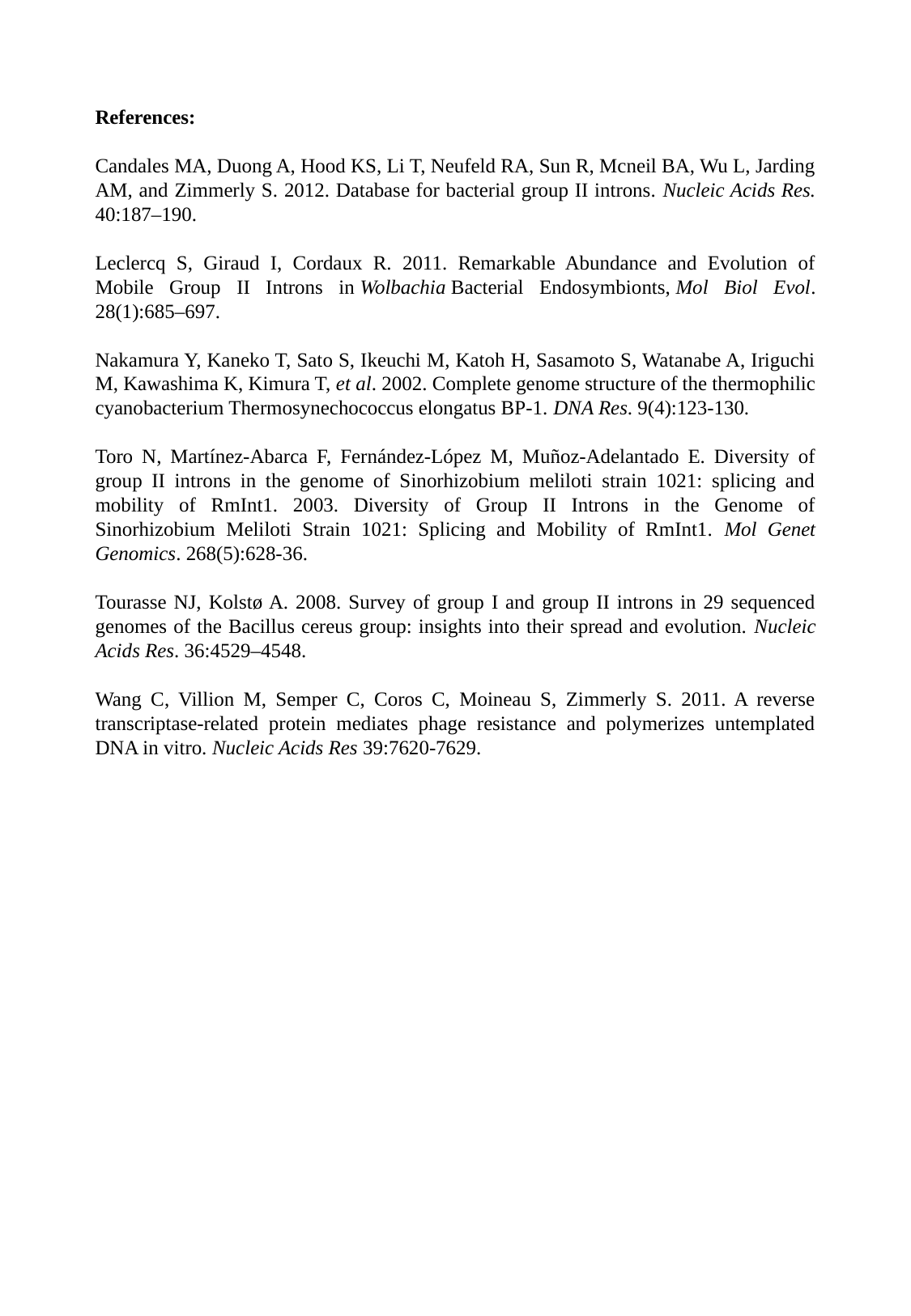

References:
Candales MA, Duong A, Hood KS, Li T, Neufeld RA, Sun R, Mcneil BA, Wu L, Jarding AM, and Zimmerly S. 2012. Database for bacterial group II introns. Nucleic Acids Res. 40:187–190.
Leclercq S, Giraud I, Cordaux R. 2011. Remarkable Abundance and Evolution of Mobile Group II Introns in Wolbachia Bacterial Endosymbionts, Mol Biol Evol. 28(1):685–697.
Nakamura Y, Kaneko T, Sato S, Ikeuchi M, Katoh H, Sasamoto S, Watanabe A, Iriguchi M, Kawashima K, Kimura T, et al. 2002. Complete genome structure of the thermophilic cyanobacterium Thermosynechococcus elongatus BP-1. DNA Res. 9(4):123-130.
Toro N, Martínez-Abarca F, Fernández-López M, Muñoz-Adelantado E. Diversity of group II introns in the genome of Sinorhizobium meliloti strain 1021: splicing and mobility of RmInt1. 2003. Diversity of Group II Introns in the Genome of Sinorhizobium Meliloti Strain 1021: Splicing and Mobility of RmInt1. Mol Genet Genomics. 268(5):628-36.
Tourasse NJ, Kolstø A. 2008. Survey of group I and group II introns in 29 sequenced genomes of the Bacillus cereus group: insights into their spread and evolution. Nucleic Acids Res. 36:4529–4548.
Wang C, Villion M, Semper C, Coros C, Moineau S, Zimmerly S. 2011. A reverse transcriptase-related protein mediates phage resistance and polymerizes untemplated DNA in vitro. Nucleic Acids Res 39:7620-7629.
